# ECG-based estimation of respiration-induced autonomic modulation of AV nodal conduction during atrial fibrillation

**DOI:** 10.3389/fphys.2024.1281343

**Published:** 2024-05-08

**Authors:** Felix Plappert, Gunnar Engström, Pyotr G. Platonov, Mikael Wallman, Frida Sandberg

**Affiliations:** ^1^ Department of Biomedical Engineering, Lund University, Lund, Sweden; ^2^ Department of Clinical Sciences, Cardiovascular Research–Epidemiology, Malmö, Sweden; ^3^ Department of Cardiology, Clinical Sciences, Lund University, Lund, Sweden; ^4^ Fraunhofer-Chalmers Centre, Department of Systems and Data Analysis, Gothenburg, Sweden

**Keywords:** atrial fibrillation, atrioventricular node, autonomic nervous system dysfunction, respiration-induced autonomic modulation, convolutional neural network, deep breathing test, network model, ECG

## Abstract

**Introduction:** Information about autonomic nervous system (ANS) activity may offer insights about atrial fibrillation (AF) progression and support personalized AF treatment but is not easily accessible from the ECG. In this study, we propose a new approach for ECG-based assessment of respiratory modulation in atrioventricular (AV) nodal refractory period and conduction delay.

**Methods:** A 1-dimensional convolutional neural network (1D-CNN) was trained to estimate respiratory modulation of AV nodal conduction properties from 1-minute segments of RR series, respiration signals, and atrial fibrillatory rates (AFR) using synthetic data that replicates clinical ECG-derived data. The synthetic data were generated using a network model of the AV node and 4 million unique model parameter sets. The 1D-CNN was then used to analyze respiratory modulation in clinical deep breathing test data of 28 patients in AF, where an ECG-derived respiration signal was extracted using a novel approach based on periodic component analysis.

**Results:** We demonstrated using synthetic data that the 1D-CNN can estimate the respiratory modulation from RR series alone with a Pearson sample correlation of *r* = 0.805 and that the addition of either respiration signal (*r* = 0.830), AFR (*r* = 0.837), or both (*r* = 0.855) improves the estimation.

**Discussion:** Initial results from analysis of ECG data suggest that our proposed estimate of respiration-induced autonomic modulation, *a*_*resp*_, is reproducible and sufficiently sensitive to monitor changes and detect individual differences. However, further studies are needed to verify the reproducibility, sensitivity, and clinical significance of *a*_*resp*_.

## 1 Introduction

Atrial fibrillation (AF) is the most common supraventricular tachyarrhythmia (Hindricks et al., 2020). Characteristic of AF is an uncoordinated atrial electrical activation that results in increased and irregular ventricular activity. Atrial fibrillation poses a significant burden to patients, physicians, and healthcare systems globally, and is associated with substantial morbidity and mortality. The recently updated guideline for the diagnosis and management of AF emphasizes that AF is a progressive disease that requires a variety of strategies at different stages, from prevention, lifestyle and risk factor modification, screening and therapy (Joglar et al., 2023). In this context, monitoring of pathophysiological changes associated with AF progression in individual patients can be valuable for the management of persistent AF.

There is a bidirectional relationship between AF and autonomic nervous system (ANS) dysfunction (Linz et al., 2019; Malik et al., 2022). The ANS contributes to the maintenance of AF (Shen and Zipes, 2014; Joglar et al., 2023) and the presence of AF promotes atrial neural remodeling and deficiencies in autonomic afferent reflexes (Wasmund et al., 2003; Yu et al., 2014; Malik et al., 2022). For example, AF patients have shown impaired sensitivity in the arterial baroreceptor reflex, a mechanism that buffers acute changes in arterial blood pressure by modulating both the parasympathetic and sympathetic nervous systems (van den Berg et al., 2001; Miyoshi et al., 2020; Ferreira et al., 2023). Conversely, the restoration of sinus rhythm has been shown to improve the baroreceptor sensitivity (Field et al., 2016), and baroreceptor activation therapy has restored sinus rhythm in a recent case study (Wang et al., 2023).

In normal sinus rhythm (NSR), autonomic dysfunction can be assessed by measuring the heart rate variability (Sassi et al., 2015; Shaffer and Ginsberg, 2017), quantifying autonomic modulation of the sinoatrial (SA) node. However, during AF, the heart rate is instead determined by the rate of fibrillation and the subsequent atrioventricular (AV) nodal modulation, raising the need for alternative approaches to assess autonomic dysfunction. Since the AV node, much like the SA node, is densely innervated by the ANS (George et al., 2017; Hanna et al., 2021), it is an attractive substitute for the assessment of autonomic function under AF. However, the relation between cardiac ANS modulation and AV nodal function under AF is far more complex than that between ANS modulation and SA node function during NSR. This calls for more sophisticated, model-based methods of analysis.

The AV node is characterized by its dual-pathway physiology allowing for parallel conduction of impulses where the two pathways have different electrophysiological properties (George et al., 2017). The fast pathway (FP) exhibits a shorter conduction delay and longer refractory period compared to the slow pathway (SP) (George et al., 2017). The AV nodal refractory period and conduction delay are influenced by the previous activity of conducted and blocked impulses (George et al., 2017; Billette and Tadros, 2019). There have been several AV node models proposed that describe different characteristics of the AV nodal structure and electrophysiology (Cohen et al., 1983; Mangin et al., 2005; Rashidi and Khodarahmi, 2005; Lian et al., 2006; Climent et al., 2011b; Masè et al., 2015; Henriksson et al., 2016; Inada et al., 2017; Wallman and Sandberg, 2018; Karlsson et al., 2021), but our previously proposed model (Plappert et al., 2022) is the first to address autonomic modulation of the AV nodal refractory period and conduction delay. We showed that ANS-induced changes during tilt could be better replicated when scaling the refractory period and conduction delay with a constant factor. Because respiration is a powerful modulator of the reflex control systems, to a large extent via effects on the baroreflex (Piepoli et al., 1997), abnormal respiration-induced autonomic modulation is often an early sign of autonomic dysfunction (Bernardi et al., 2001). For the monitoring of cardiac autonomic modulation in AF patients, the assessment of respiration-induced autonomic modulation seems well-suited because respiration is always present and can be extracted from ECG signals (Varon et al., 2020). Building on the previous AV node model extension, the respiration-induced autonomic modulation could be incorporated by time-varying changes in the modulation of AV nodal refractory period and conduction delay.

Machine learning is vibrant in the field of cardiac electrophysiology with a rapidly growing number of applications (Trayanova et al., 2021). However, one main challenge is the acquirement of large amounts of data for proper training and validation. In recent years, a few studies have been performed in which synthetic data has been generated for the training of neural networks which are then used on clinical data. For example, synthetic images were generated to train neural networks to track cardiac motion and calculate cardiac strain (Loecher et al., 2021), estimate tensors from free-breathing cardiac diffusion tensor imaging (Weine et al., 2022), and predict end-diastole volume, end-systole volume, and ejection fraction (Gheorghita et al., 2022). Furthermore, synthetic photoplethysmography (PPG) signals were generated to detect bradycardia and tachycardia (Sološenko et al., 2022), and synthetic electrocardiogram (ECG) signals were generated to detect r-waves during different physical activities and atrial fibrillation (Kaisti et al., 2023), and to predict the ventricular origin in outflow tract ventricular arrhythmias (Doste et al., 2022).

This study aims to develop and evaluate a method to extract respiration-induced autonomic modulation in the AV node conduction properties from ECG data in AF. We present a novel approach to extract respiration signals from several ECG leads based on the periodic component analysis (Sameni et al., 2008). In addition, we present a novel extension to our previously proposed AV node network model accounting for respiration-induced autonomic modulation of AV nodal refractory period and conduction delay. Furthermore, we estimate the magnitude of respiration-induced autonomic modulation using a 1-dimensional convolutional neural network that was trained on synthetic 1-min segments of RR series, respiration signals, and average atrial fibrillatory rate which replicate clinical data. The trained network was used to analyze data from 28 AF patients performing a deep breathing task including slow metronome breathing at a respiration rate of 6 breaths/min. During NSR, slower breathing causes an increased respiration-induced autonomic modulation with a maximum HRV response typically observed at a respiration rate of 6 breaths/min (Russo et al., 2017). Hence, we hypothesize that the respiration-induced autonomic modulation in the AV node conduction properties is strengthened during the deep breathing task.

## 2 Materials and methods

First, the clinical deep breathing test data from patients in atrial fibrillation is described in Section 2.1. In Section 2.2, the extraction of RR series and atrial fibrillatory rate (AFR) from ECG are described. Moreover, Section 2.2 covers the extraction of ECG-derived respiration (EDR) signals using a novel approach based on periodic component analysis. A description of the extended AV network model accounting for respiration-induced autonomic modulation is given in Section 2.3, as well as a description of how the simulated datasets are generated. In Section 2.4, the architecture of a 1-dimensional convolutional neural network (1D-CNN) that is used to estimate the magnitude of respiratory modulation from ECG recordings is described together with the training and testing of the neural network. Finally, the CNN is used to estimate the respiration-induced autonomic modulation from the clinical ECG-derived features and the estimates are analyzed.

### 2.1 ECG data

The dataset of the clinical deep breathing test consisted of 12-lead ECG recordings with a sampling rate of 500 Hz from individuals with AF participating in the SCAPIS study (Bergström et al., 2015). The participants in the SCAPIS study were from the Swedish general population aged 50–64 years. A subset of the SCAPIS cohort (5136 participants) performed a deep breathing test (Engström et al., 2022). Of this subset, 28 participants with complete data were in AF at the time of recording (Abdollahpur et al., 2022). The clinical characteristics of that subset are listed in Table 1. The deep breathing test started with the participants resting in a supine position while breathing normally for 5 minutes. Following this, the participants performed slow metronome breathing at a respiration rate of 0.1 Hz for 1 minute. During the slow metronome breathing, a nurse guided the participants to inhale for 5 seconds and exhale for 5 seconds, for a total of six breathing cycles.

**TABLE 1:** Clinical characteristics of study population.

	Number
Age	60.1 ± 4.0 [50.1-64.9]
Men	23 (82%)
BMI	31.8 ± 7.2 [18.8-50.8]
Systolic BP	124 ± 23 [90-188]
Diastolic BP	79.9 ± 11 [61-104]
Hypertension^*^	17 (61%)
Diabetes	2 (7%)
Never smokers	9 (32%)
Heart failure	2 (7%)
Previous AMI or angina	2 (7%)
Beta blocker	15 (54%)
Ca-antagonist	6 (21%)
Antiarrhythmic drug	4 (14%)

^*^ ≥140/90 mmHg or treatment for hypertension. Values are given in the following formats: number, mean ± SD, [range]; BP, blood pressure.

### 2.2 ECG data processing

#### 2.2.1 Extraction of RR series

ECG preprocessing and QRS complex detection were performed using the CardioLund ECG parser (www.cardiolund.com). The CardioLund ECG parser classified QRS complexes based on their QRS morphology. Only QRS complexes with dominant QRS morphology were considered in the computation of the RR series.

The RR series were computed from intervals between R-peaks taken from consecutive QRS complexes with dominant QRS morphology, and the time of each RR interval was set to the time of the first R-peak in each interval. The resulting non-uniformly sampled RR series were interpolated to a uniform sampling rate of 4 Hz using piecewise cubic Hermite polynomials as implemented in MATLAB (‘pchip’, version R2023a, RRID:SCR_001622).

#### 2.2.2 Estimation of atrial fibrillatory rate

The AFR was used to obtain information about the atrial arrival process. Briefly, the estimation of the AFR involved the extraction of an f-wave signal by means of spatiotemporal QRST-cancellation (Stridh and Sörnmo, 2001) and estimation of an f-wave frequency trend by fitting two complex exponential functions to the extracted f-wave signal from ECG lead V1 as proposed in (Henriksson et al., 2018). The two exponential functions were characterized by a fundamental frequency *f* and its second harmonic, respectively; *f* was fitted within the range $f_{\max}^{\mathrm{Welch}}\pm 1.5$ Hz, where $f_{\max}^{\mathrm{Welch}}$ denotes the maximum of the Welch periodogram of ECG lead V1 in the range 4–12 Hz. The results for the deep breathing data have been previously presented in (Abdollahpur et al., 2022). The estimated AFR signal has a sampling rate of 50 Hz.

#### 2.2.3 Extraction of lead-specific EDR signals

All steps of the extraction algorithm that are described in the following were applied to 1-min segments of the lead-specific EDR signals taken from a 1-min running window. The lead-specific EDR signals were computed with the slope range method (Kontaxis et al., 2020) for the eight ECG leads V1-V6, I, and II. Only eight out of 12 ECG leads were used, because the information in the leads III, aVF, aVL, and aVR can also be derived from lead I and II. The slope range method uses the peak-to-peak difference in the first derivative of the QRS complex to quantify the variations in the QRS morphology that are assumed to reflect the respiratory activity and are caused, for example, by periodic changes in electrode positions relative to the heart.

Only QRS complexes with dominant QRS morphology (cf. Section 2.2.1) were considered when applying the slope-range method. Further, a QRS complex was excluded as an outlier from analysis if the slope range value of any of the leads was outside the mean ± 3 std of the slope range values of that lead. The lead-specific non-uniformly sampled EDR signals were interpolated to a uniform sampling rate of 4 Hz using the modified Akima algorithm as implemented in MATLAB (‘makima’, version R2023a, RRID:SCR_001622). A matrix containing the resampled lead-specific EDR signals $X^{\prime }={\left[x_{1}^{\prime },\ldots ,x_{8}^{\prime }\right]}^{T}$ of dimension 8 × *N* was constructed, where *N* = 240 corresponds to the length of the 1-min segment. To remove baseline-wander in **X**′, a Butterworth highpass filter of order 4 with a cut-off frequency of 0.08 Hz was applied separately for each lead **x**′. The filtered **X**′ was normalized to zero-mean and signals shorter than 1 min were zero-padded to create **X** containing 1-min segments. A set $S_{\mathrm{seg}}$ was created containing all **X**_*i*_, where *i* = 1, … , *I* denotes all *I* possible choices of 1-min segments of the lead-specific EDR signals from one recording.

Algorithm 1 Extraction of joint-lead EDR signals.

**for** all **X**_*i*_ in $S_{\mathrm{seg}}$ **do**
 **X**_*i*_ is whitened according to Eq. 1 to obtain **Z**_*i*_
 **for** all *τ*_*j*_∈[10, 40] **do**
  obtain **w**_*j*_ by solving the generalized eigenvalue problem of matrix pair $\left({\bar{C}}_{z}\left(\tau_{j}\right),{\bar{C}}_{z}\left(0\right)\right)$

  compute *ϵ*(**w**_*j*_, *τ*_*j*_, **Z**_*i*_) according to Eq. 2
 **end** **for**
**end** **for**
compute $\tau^{*}=\min_{\tau_{j}}\left(\sum_{S_{\mathrm{seg}}}\epsilon \left(w_{j},\tau_{j},Z_{i}\right)\right)$

**for** all **Z**_*i*_ in $S_{\mathrm{seg}}$ **do**
 
$S_{\tau }=\emptyset$

 **for** all *τ*_*j*_∈[10, 40] **do**
  **if** *ϵ*(**w**_*j*_, *τ*_*j*_, **Z**_*i*_) ≤ *ϵ*(**w**_*j*_, *τ*_*j*−1_, **Z**_*i*_) ∨ *τ*_*j*_= = 10 **then**
   **if** *ϵ*(**w**_*j*_, *τ*_*j*_, **Z**_*i*_) ≤ *ϵ*(**w**_*j*_, *τ*_*j*+1_, **Z**_*i*_) ∨ *τ*_*j*_= = 40 **then**
    add *τ*_*j*_ to $S_{\tau }$

   **end** **if**
  **end** **if**
 **end** **for**
 set *τ*_*resp*_ as value in $S_{\tau }$ closest to *τ* ^*^
 obtain **w**_*resp*_ by solving the generalized eigenvalue problem of matrix pair $\left({\bar{C}}_{z}\left(\tau_{\mathrm{resp}}\right),{\bar{C}}_{z}\left(0\right)\right)$

 
$s_{i}^{*}=w_{\mathrm{resp}}^{T}Z_{i}\cdot sign\left(\sum w_{\mathrm{resp}}\right)$

 *f*_*resp,i*_= *f*_*s*_/*τ*_*resp*_
**end** **for**

#### 2.2.4 Extraction of joint-lead EDR signals

The joint-lead EDR signal was extracted from **X** using a modified version of the periodic component analysis (*π*CA) (Sameni et al., 2008), summarized in Algorithm 1. The matrix **X** was whitened for its elements to be uncorrelated and to have unit variance. The whitened lead-specific EDR signals **Z** were computed as

$$Z=D^{-1/2}E^{T}X,$$ (1)

where **D** is the diagonal matrix of eigenvalues of the covariance matrix $C_{X}=E\left\{XX^{T}\right\}$ , and the columns of the matrix **E** are the unit-norm eigenvectors of **C**_**X**_.

The outputs of the *π*CA are a joint-lead EDR signal **s** of dimension 1 × *N* and its corresponding lag *τ*. The assumption of the *π*CA is that $s=w^{T}Z$ is a linear mixture of the whitened lead-specific EDR signals. The aim is to find a solution for **s** with a maximal periodic structure. The periodic structure of **s** is characterized by *ϵ*(**w**, *τ*, **Z**), which quantifies non-periodicity (Sameni et al., 2008) and is defined as

$$\epsilon \left(w,\tau ,Z\right)=\frac{\sum_{n}|s\left(n+\tau \right)-s\left(n\right){|}^{2}}{\sum_{n}|s\left(n\right){|}^{2}}=2\left[1-\frac{w^{T}{\bar{C}}_{z}\left(\tau \right)w}{w^{T}{\bar{C}}_{z}\left(0\right)w}\right],$$ (2)

where *s*(*n*) is the *n*:th element of **s**. We solved the generalized eigenvalue problem (GEP) of the lag-dependent matrix pair $\left({\bar{C}}_{z}\left(\tau \right),{\bar{C}}_{z}\left(0\right)\right)$ to obtain a full matrix **V** whose columns correspond to the right eigenvectors and a diagonal matrix **U** of generalized eigenvalues so that ${\bar{C}}_{z}\left(\tau \right)V={\bar{C}}_{z}\left(0\right)VU$ (Sameni et al., 2008). Here, ${\bar{C}}_{z}\left(\tau \right)=[C_{z}\left(\tau \right)+{\left(C_{z}\left(\tau \right)\right)}^{T}+C_{z}\left(-\tau \right)+{\left(C_{z}\left(-\tau \right)\right)}^{T}]/4$ for some lag *τ* is a modified lagged covariance matrix, which is always symmetric, unlike the time lagged covariance matrix $C_{z}\left(\tau \right)=E_{n}\left\{z\left(n\right)z{\left(n-\tau \right)}^{T}\right\}$ , where **z**(*n*) is the *n*:th column of **Z** and *E*_*n*_{⋅} indicates averaging over *n*. The weight vector $w={\left[w_{1},\ldots ,w_{8}\right]}^{T}$ that minimizes *ϵ*(**w**, *τ*, **Z**) is obtained as the first column of **V** (Sameni et al., 2008). In the present study, *ϵ*(**w**, *τ*, **Z**) is also used to quantify signal quality, where a lower value of *ϵ*(**w**, *τ*, **Z**) corresponds to a more periodic signal assumed to have a higher SNR.

As *τ* is unknown, *ϵ*(**w**, *τ*, **Z**) was minimized for all integer values of *τ* between 10 and 40, corresponding to respiration rates between 0.1 and 0.4 Hz. To improve the robustness of the *π*CA for signals with low quality, a *τ* ^*^ was determined in an intermediate step that corresponds to a global minimum of *ϵ*(**w**, *τ*, **Z**) over all 1-min segments in $S_{\mathrm{seg}}$ . It was assumed that there were no significant transient changes in respiration frequency in the clinical data and we determined two different *τ* ^*^ for each subject; one for normal breathing and one for deep breathing. Then, for each 1-min segment separately, a *τ*_*resp*_ was estimated as the local minimum of *ϵ*(**w**, *τ*, **Z**) closest to *τ* ^*^. The respiration frequency estimate f^resp=fs/τ^resp results from the estimate ${\hat{\tau }}_{\mathrm{resp}}$ and the sampling rate *f*_*s*_ = 4 Hz and is in the range f^resp∈[0.1,0.4] Hz corresponding to the limits set by *τ*. Finally, the weight vector **w**_*resp*_ for the respiration signal extraction was obtained by solving the GEP of the matrix pair $\left({\bar{C}}_{z}\left(\tau_{\mathrm{resp}}\right),{\bar{C}}_{z}\left(0\right)\right)$ . The extracted $s=w_{\mathrm{resp}}^{T}Z$ was normalized to unit variance. An ambiguity of *π*CA is that the sign of **s** is undetermined. The sign of the joint-lead EDR signal was selected as $s^{*}=s\cdot sign\left(\sum w_{\mathrm{resp}}\right)$ , where *∑* **w**_*resp*_ denotes the sum of the elements in the vector **w**_*resp*_. This was done under the assumption that all lead-specific EDR signals are in phase.

#### 2.2.5 Estimates from clinical data

The joint-lead EDR signal extraction from Section 2.2.4 was applied to all 1-min segments **X** in $S_{\mathrm{seg}}$ for each patient and recording. Segments **X** were excluded from further analysis if they do not satisfy the following three criteria, for which a valid QRS complex has a dominant QRS morphology and is not classified as outlier based on its slope range values: *i*) the maximum distance between valid QRS complexes is 2 s; *ii*) the minimum number of valid QRS complexes in a 1-min segment is 48; *iii*) the minimum number of valid QRS complexes in a 1-min segment is 80% of the normal-to-normal average heart rate of the 1-min segment. After exclusion, several sets of non-overlapping 1-min segments could be created from the remaining **X**. Out of these, the set $S_{\mathrm{seg}}^{*}$ that resulted in the smallest sum of *ϵ*(**w**_*resp*_, *τ*_*resp*_, **Z**) was chosen, and used to produce joint-lead EDR signals $X_{\mathrm{Resp}}^{\mathrm{Clin}}$ of dimension 1 × *N* as described in Section 2.2.4. In addition, the corresponding 1-min RR series $X_{RR}^{\mathrm{Clin}}$ of dimension 1 × *N* was extracted from the RR series obtained in Section 2.2.1. We estimated the mean arrival rate of atrial impulses to the AV node $\hat{\mu }$ as $1000/\bar{AFR}$ , where $\bar{AFR}$ is the average AFR-trend within each of the selected 1-min windows as described in Section 2.2.2. To match the dimensions of $X_{RR}^{\mathrm{Clin}}$ and $X_{\mathrm{Resp}}^{\mathrm{Clin}}$ , $\hat{\mu }$ was then repeated *N* times to produce $X_{\mathrm{AFR}}^{\mathrm{Clin}}$ of dimension 1 × *N*. From the clinical data, a maximum of five non-overlapping 1-min long segments in normal breathing and one segment in deep breathing was obtained for $X_{RR}^{\mathrm{Clin}}$ , $X_{\mathrm{Resp}}^{\mathrm{Clin}}$ and $X_{\mathrm{AFR}}^{\mathrm{Clin}}$ .

### 2.3 Simulated data

#### 2.3.1 Network model of the human atrioventricular node

**FIGURE 1:**
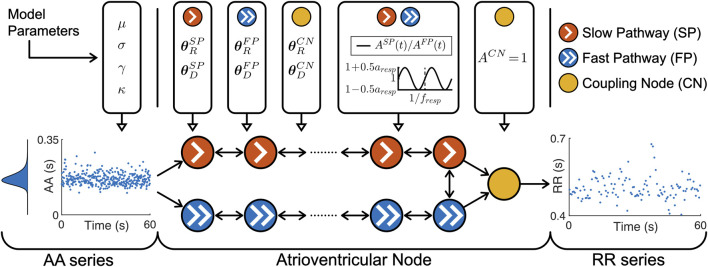
A schematic representation of the AV node model. Retrograde conduction was possible within the AV node model. For simplicity, only a subset of the ten nodes in each pathway is shown. Note that the atrioventricular node used different $\theta_{R}^{P}$ and $\theta_{D}^{P}$ for the three different pathways, the same time-varying *A* ^*P*^(*t*) for SP and FP and a constant *A* ^*CN*^ = for CN.

The atrioventricular node is modeled by a network of 21 nodes (cf. Figure 1). The presented AV node model was initially proposed in (Wallman and Sandberg, 2018), updated with minor modifications in (Karlsson et al., 2021), and extended using constant scaling factors *A*_*R*_ and *A*_*D*_ for the refractory period and conduction delay to account for the effect of changes in autonomic modulation in (Plappert et al., 2022). The slow pathway (SP) and fast pathway (FP) are described by two chains of 10 nodes each, which are only connected at their last nodes. Impulses enter the AV node model simultaneously at the first node of each pathway. Within the pathways and between their last nodes, the impulses are conducted bidirectionally to allow for retrograde conduction. The last nodes of the two pathways are connected to an additional coupling node (CN), through which the impulses leave the model.

Each node represents a section of the AV node and is characterized by an individual refractory period $R^{P}\;\left(\Delta t_{k},A^{P}\left(t\right),\theta_{R}^{P}\right)$ and conduction delay $D^{P}\;\left(\Delta t_{k},A^{P}\left(t\right),\theta_{D}^{P}\right)$ defined as

$$R^{P}\;\left(\Delta t_{k},A^{P}\left(t\right),\theta_{R}^{P}\right)=A^{P}\left(t\right)\left(R_{\min}^{P}+\Delta R^{P}\left(1-e^{-\Delta t_{k}/\tau_{R}^{P}}\right)\right)$$ (3)

$$D^{P}\;\left(\Delta t_{k},A^{P}\left(t\right),\theta_{D}^{P}\right)=A^{P}\left(t\right)\left(D_{\min}^{P}+\Delta D^{P}e^{-\Delta t_{k}/\tau_{D}^{P}}\right)$$ (4)

Where *P* ∈ {*SP*, *FP*, *CN*} denotes the pathway. The refractory period and conduction delay are defined by fixed model parameters for the refractory period $\theta_{R}^{P}$ and conduction delay $\theta_{D}^{P}$ as well as model states for the diastolic interval Δ*t*_*k*_ and respiratory modulation *A* ^*P*^(*t*). Each pathway has a separate set of fixed model parameters for the refractory period $\theta_{R}^{P}=$ [ $R_{\min}^{P}$ , Δ*R* ^*P*^, $\tau_{R}^{P}$ ] and conduction delay $\theta_{D}^{P}=$ [ $D_{\min}^{P}$ , Δ*D* ^*P*^, $\tau_{D}^{P}$ ], where $R_{\min}^{P}$ is the minimum refractory period, Δ*R* ^*P*^ is the maximum prolongation of the refractory period, $\tau_{R}^{P}$ is a time constant, $D_{\min}^{P}$ is the minimum conduction delay, Δ*D* ^*P*^ is the maximum prolongation of the conduction delay and $\tau_{D}^{P}$ is a time constant. For clarity, the notation of *R* ^*P*^ (⋅, *A* ^*P*^(*t*), ⋅) and *D* ^*P*^ (⋅, *A* ^*P*^(*t*), ⋅) are specified with dots when the replaced parameters or model states are currently not discussed.

The scaling factor *A* ^*P*^(*t*) accounts for the effect of changes in autonomic modulation on the refractory period $R^{P}\;\left(\cdot ,A^{P}\left(t\right),\cdot \right)$ and the conduction delay $D^{P}\;\left(\cdot ,A^{P}\left(t\right),\cdot \right)$ . The time-varying scaling factor *A* ^*P*^(*t*) is common between the SP and FP, defined in Eq. 5 as

$$A^{SP}\left(t\right)=A^{FP}\left(t\right)=1+\frac{a_{\mathrm{resp}}}{2}\;sin\left(2\pi tf_{\mathrm{resp}}\right),$$ (5)

with a constant respiratory frequency *f*_*resp*_ and peak-to-peak amplitude *a*_*resp*_. The scaling factor of the refractory period and conduction delay of the CN is described by *A* ^*CN*^ = 1 and not modulated by respiration.

The electrical excitation propagation through the AV node is modeled as a series of impulses that can either be conducted or blocked by a node. An impulse is conducted to all adjacent nodes, if the interval Δ*t*_*k*_ between the *k*:th impulse arrival time *t*_*k*_ and the end of the (*k*–1):th refractory period, computed as

$$\Delta t_{k}=t_{k}-t_{k-1}-R^{P}\;\left(\Delta t_{k-1},\cdot ,\cdot \right)$$ (6)

is positive. Then, the time delay between the arrival of an impulse at a node and its transmission to all adjacent nodes is given by the conduction delay $D^{P}\;\left(\Delta t_{k},\cdot ,\cdot \right)$ . If Δ*t*_*k*_ is negative, the impulse is blocked due to the ongoing refractory period $R^{P}\;\left(\Delta t_{k-1},\cdot ,\cdot \right)$ . After an impulse is conducted, $R^{P}\;\left(\Delta t_{k},\cdot ,\cdot \right)$ and $D^{P}\;\left(\Delta t_{k},\cdot ,\cdot \right)$ of the current node are updated according to Eqs 3, 4, 6. Details about how the impulses are processed chronologically and node by node, using a priority queue of nodes and sorted by impulse arrival time, can be found in (Wallman and Sandberg, 2018).

The input to the AV node mode is a series of atrial impulses during AF, with inter-arrival times modeled according to a Pearson Type IV distribution (Climent et al., 2011a). The AA series is generated with a point process with independent inter-arrival times and is completely characterized by the four parameters of the Pearson Type IV distribution, namely, the mean *μ*, standard deviation *σ*, skewness *γ* and kurtosis *κ*.

The output of the AV node model is a series of ventricular impulses, where $t_{q}^{V}$ denotes the time of the *q*:th ventricular impulse. As the refractory period $R^{P}\;\left(\Delta t_{k},\cdot ,\cdot \right)$ and conduction delay $D^{P}\;\left(\Delta t_{k},\cdot ,\cdot \right)$ are history-dependent, the first 1,000 ventricular impulses leaving the AV node model are excluded from analysis to avoid transient effects.

#### 2.3.2 Simulation of AV nodal conduction

For the training and validation, a dataset with 2 million unique parameter sets was generated. This dataset was divided into 20 datasets with 100,000 parameter sets each, where a dataset was either used for training or validation of one of ten realizations of the convolutional neural network (CNN) that is described in Section 2.4.2. Simulations were performed with each parameter set using the AV node model described in Section 2.3.1. For each simulation, a series of 11,000 AA intervals was generated using the Pearson Type IV distribution, defined by the four parameters *μ*, *σ*, *γ*, and *κ*. The parameter *μ* was randomly drawn from U[100,250] ms and *σ* was randomly drawn from U[15,30] ms. The parameters *γ* and *κ* were kept fixed at 1 and 6, respectively, since they cannot be estimated from the f-waves of the ECG (Plappert et al., 2022). Negative AA intervals were excluded from the impulse series. The model parameters for the refractory period $\theta_{R}^{P}$ and conduction delay $\theta_{D}^{P}$ of the SP and FP were drawn from bounded uniform distributions and the model parameters of the CN were kept fixed according to Table 2. The given ranges were in line with our previous work (Plappert et al., 2022). The model parameters for the respiration-induced autonomic modulation and simulated respiration signal that are used in Section 2.3.3 were also drawn from bounded uniform distributions, with *a*_*resp*_ randomly drawn from U[−0.1,0.5] , *f*_*resp*_ randomly drawn from U[0.1,0.4] Hz and *η* randomly drawn from U[0.2,4] . For testing, another dataset with 2 million unique parameter sets was generated using the same ranges listed above, except for *a*_*resp*_, which was randomly drawn from U[0,0.4] .

**TABLE 2:** AV Node model parameters used for simulated data.

Parameters		P ≡SP (ms)	P ≡FP (ms)	P ≡CN (ms)
$\theta_{R}^{P}$	$R_{\min}^{P}$	U[250,600]	U[250,600]	250
	Δ*R* ^*P*^	U[0,600]	U[0,600]	0
	$\tau_{R}^{P}$	U[50,300]	U[50,300]	1
$\theta_{D}^{P}$	$D_{\min}^{P}$	U[0,30]	U[0,30]	0
	Δ*D* ^*P*^	U[0,75]	U[0,75]	0
	$\tau_{D}^{P}$	U[50,300]	U[50,300]	1

When sampling, initially a value for *a*_*resp*_ was drawn from a uniform distribution. To exclude non-physiological parameter sets from the dataset, we resampled the rest of the parameters until the following five selection criteria were met: 1) the slow pathway in every parameter set must have a higher conduction delay $D^{SP}\;\left(\Delta t_{k},\cdot ,\cdot \right)>D^{FP}\;\left(\Delta t_{k},\cdot ,\cdot \right)$ and lower refractory period $R^{SP}\;\left(\Delta t_{k},\cdot ,\cdot \right)<R^{FP}\;\left(\Delta t_{k},\cdot ,\cdot \right)$ than the fast pathway for all Δ*t*_*k*_; 2) the resulting average RR interval has to fall within the range of 300 ms–1,000 ms, which corresponds to heart rates between 60 bpm and 200 bpm; 3) the resulting root mean square of successive RR interval differences (RR RMSSD) has to be above 100 ms; 4) the resulting sample entropy of the RR series has to be above 1; 5) the relative contribution of the respiration frequency in the frequency spectrum of the RR series with zero-mean *F*_*RR*_(*f*_*resp*_)/*∑*_*f*_ *F*_*RR*_(*f*) has to be below 7% to exclude RR series with visible periodicity. Note that the frequency spectrum is computed from the RR series with 240 samples and the sampling rate of 4 Hz.

Similar to the clinical data described in Section 2.2.1, RR series were computed from intervals between the simulated ventricular impulses, and the time of each RR interval sample was set to the time of the first ventricular impulse. The resulting non-uniformly sampled RR series were interpolated to a uniform sampling rate of 4 Hz using piecewise cubic hermite interpolating polynomials as implemented in MATLAB (‘pchip’, version R2023a, RRID:SCR_001622). The simulated RR series were cut into 1-min segments of length *N* = 240, resulting in RR series $X_{RR}^{\mathrm{Sim}}$ of dimension 1 × *N*. For each RR series, *μ* was repeated *N* times to form a vector $X_{\mathrm{AFR}}^{\mathrm{Sim}}$ of dimension 1 × *N*, corresponding to the mean atrial arrival rate.

#### 2.3.3 Modelling respiratory signals

For the modeling of the respiratory signals resembling joint-lead EDR signals (cf. Section 2.2.4), we start with the underlying assumption that respiration can be described according to *m*(*t*) = sin(2*πtf*_*resp*_), i.e., by a sine wave oscillating at the respiratory frequency *f*_*resp*_. Eight identical lead-specific EDR signals $m_{p}^{\prime }\left(t\right)$ with *p* = 1, … , 8 were created, composed of non-uniform samples of *m*(*t*) at the times of the ventricular impulses $t_{q}^{V}$ generated by the AV node model. To emulate lead-specific EDR signals, Gaussian noise with standard deviation *η* was added to all samples of $m_{p}^{\prime }\left(t\right)$ , making them non-identical.

Next, $m_{p}^{\prime }\left(t\right)$ were processed in five steps to mimic the processing steps for the clinical ECG-derived features (cf. Sections 2.2.3 and 2.2.4): 1) using the same criteria as for the outlier exclusion in the clinical data, all samples in $m_{p}^{\prime }\left(t\right)$ for the same ventricular impulse were excluded as outliers, if the value in one of the eight leads was outside the mean ± 3 std, computed for each lead within a 1-min running window; 2) as for the clinical lead-specific EDR signals, the simulated signals $m_{p}^{\prime }\left(t\right)$ were interpolated to a uniform sampling rate of 4 Hz using the modified Akima algorithm as implemented in MATLAB (‘makima’, version R2023a, RRID:SCR_001622), resulting in $m_{p}^{\prime }\left(n\right)$ ; 3) $m_{p}^{\prime }\left(n\right)$ were cut into 1-min segments of length *N* = 240 and had the dimension 8 × *N*; 4) the resampled and cut signals are filtered with a Butterworth highpass filter of order 4 with the cut-off frequency 0.08 Hz to remove baseline-wander; 5) a joint-lead EDR signal $X_{\mathrm{Resp}}^{\mathrm{Sim}}$ with dimension 1 × *N* was extracted from $m_{p}^{\prime }\left(n\right)$ using the periodic component analysis described in Section 2.2.4.

### 2.4 Estimation of respiratory modulation

#### 2.4.1 Training and estimation using a linear regression model

A linear regression model is used here to estimate the peak-to-peak amplitude of respiration-induced autonomic modulation *a*_*resp*_. The linear regression model $L_{RR,Resp,AFR}$ was trained using a training dataset $X^{\mathrm{Sim,Train}}$ with the format $X=\left[X_{RR}^{\mathrm{Sim}};X_{\mathrm{Resp}}^{\mathrm{Sim}};X_{\mathrm{AFR}}^{\mathrm{Sim}}\right]$ containing 100,000 parameter sets, as described in Section 2.3.2. The performance of $L_{RR,Resp,AFR}$ on simulated data was assessed using the testing dataset $X^{\mathrm{Sim,Test}}$ containing 2 million parameter sets, as described in Section 2.3.2. The performance on $X^{\mathrm{Sim,Test}}$ was assessed using the RMSE, Pearson correlation, and coefficient of determination *R* ^2^ between the true *a*_*resp*_ and estimated ${\hat{a}}_{\mathrm{resp}}$ .

#### 2.4.2 Architecture of 1-dimensional convolutional neural network

**FIGURE 2:**
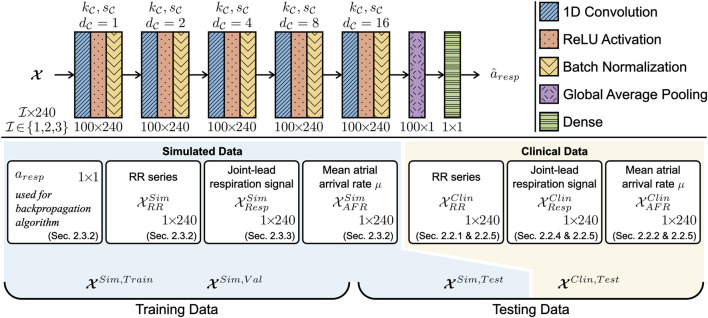
The CNN was composed of five 1D convolution layers with 100 filters each. The convolution layers had a kernel size $k_{C}$ , stride $s_{C}$ and dilation factor $d_{C}$ . Training datasets $X^{\mathrm{Sim,Train}}$ , validation datasets $X^{\mathrm{Sim},\mathrm{Val}}$ , and testing datasets $X^{\mathrm{Sim,Test}}$ were constructed from the simulated data $X_{RR}^{\mathrm{Sim}}$ , $X_{\mathrm{Resp}}^{\mathrm{Sim}}$ and $X_{\mathrm{AFR}}^{\mathrm{Sim}}$ . A testing dataset $X^{\mathrm{Clin,Test}}$ was constructed from the clinical ECG-derived features $X_{RR}^{\mathrm{Clin}}$ , $X_{\mathrm{Resp}}^{\mathrm{Clin}}$ and $X_{\mathrm{AFR}}^{\mathrm{Clin}}$ .

To estimate the peak-to-peak amplitude of the respiration-induced autonomic modulation, *a*_*resp*_, a 1D-CNN architecture was used as illustrated in Figure 2. The CNN architecture consists of five convolution layers, where each layer *l* was composed of 100 1D-CNN filters with kernel size $k_{C}=3$ , stride $s_{C}=1$ and dilation factor $d_{C}=2^{l-1}$ , followed by a rectified linear unit (RELU) and a batch normalization layer. After the five convolution layers, the data passed through a global average pooling layer and dense layer, the output of which is an estimation ${\hat{a}}_{\mathrm{resp}}$ . To assess the performance of the CNN with or without the RR series, respiration signal, and mean *μ* of the AA series, seven versions of the CNN were trained. The respective CNNs and their input data are given as follows: the CNN $C_{RR}$ was trained on the input data with the format $X=X_{RR}^{\mathrm{Sim}}$ ; $C_{\mathrm{Resp}}$ was trained on $X=X_{\mathrm{Resp}}^{\mathrm{Sim}}$ ; $C_{\mathrm{AFR}}$ was trained on $X=X_{\mathrm{AFR}}^{\mathrm{Sim}}$ ; $C_{RR,Resp}$ was trained on $X=\left[X_{RR}^{\mathrm{Sim}};X_{\mathrm{Resp}}^{\mathrm{Sim}}\right]$ ; $C_{RR,AFR}$ was trained on $X=\left[X_{RR}^{\mathrm{Sim}};X_{\mathrm{AFR}}^{\mathrm{Sim}}\right]$ ; $C_{\mathrm{Resp,AFR}}$ was trained on $X=\left[X_{\mathrm{Resp}}^{\mathrm{Sim}};X_{\mathrm{AFR}}^{\mathrm{Sim}}\right]$ ; and $C_{RR,Resp,AFR}$ was trained on $X=\left[X_{RR}^{\mathrm{Sim}};X_{\mathrm{Resp}}^{\mathrm{Sim}};X_{\mathrm{AFR}}^{\mathrm{Sim}}\right]$ .

#### 2.4.3 Training the convolutional neural network

For each CNN version, i.e., $C_{RR}$ , $C_{\mathrm{Resp}}$ , $C_{\mathrm{AFR}}$ , $C_{RR,Resp}$ , $C_{RR,AFR}$ , $C_{\mathrm{Resp,AFR}}$ and $C_{RR,Resp,AFR}$ , described in Section 2.4.2, ten realizations were trained with unique training and validation datasets, $X^{\mathrm{Sim,Train}}$ and $X^{\mathrm{Sim,Val}}$ , respectively, containing 100,000 parameter sets each, as described in Section 2.3.2. The CNNs were trained to estimate the *a*_*resp*_ and the weights of the CNN were updated during backpropagation based on the root-mean-square error (RMSE) of the residuals. Every epoch, $X^{\mathrm{Sim,Train}}$ was randomly divided into 20 mini-batches, each containing input data for 5,000 different parameter sets. A cyclical learning rate was set for the training, where the learning rate started at 5 ⋅ 10^–3^ and was increased and decreased in a ‘zig-zag’ between [2 ⋅ 10^–3^, 3 ⋅ 10^–3^, 5 ⋅ 10^–3^, 8 ⋅ 10^–3^, 10 ⋅ 10^–3^] every time the RMSE of $X^{\mathrm{Sim},\mathrm{Val}}$ did not improve for 50 epochs (Smith, 2017). The initial learning rate and the minimum and maximum boundary values of the cyclical learning rates were determined using the ‘learning rate range test’, described in (Smith, 2017). The network was validated after every epoch. The CNN was trained until the RMSE of $X^{\mathrm{Sim},\mathrm{Val}}$ did not improve for 50 epochs for each of the five learning rates, and the network weights giving the lowest validation RMSE was chosen. The estimate ${\hat{a}}_{\mathrm{resp}}$ was computed as the average of the individual estimates of each of the ten CNN realizations.

#### 2.4.4 Estimation of respiratory modulation in simulated data

The performance of the CNN on simulated data was assessed for $C_{RR}$ , $C_{\mathrm{Resp}}$ , $C_{\mathrm{AFR}}$ , $C_{RR,Resp}$ , $C_{RR,AFR}$ , $C_{\mathrm{Resp,AFR}}$ and $C_{RR,Resp,AFR}$ , using the testing dataset $X^{\mathrm{Sim,Test}}$ described in Section 2.3.2. The total performance on $X^{\mathrm{Sim,Test}}$ was assessed using the RMSE, Pearson sample correlation, and coefficient of determination *R* ^2^ between the true *a*_*resp*_ and estimated ${\hat{a}}_{\mathrm{resp}}$ .

In addition, the performance was assessed over a range of respiration frequencies *f*_*resp*_ and characteristics of non-periodicity in the respiration signal *ϵ*(**w**, *τ*, **Z**), here denoted *ϵ*. To produce local RMSE estimates $\sigma \left(f_{\mathrm{resp}}^{\prime },\epsilon^{\prime }\right)$ for specific values $f_{\mathrm{resp}}^{\prime }$ and *ϵ*′, the following three steps were applied: 1) a squared difference ${\left(a_{\mathrm{resp}}-{\hat{a}}_{\mathrm{resp}}\right)}^{2}$ was computed for each of the 2 million parameter sets in $X^{\mathrm{Sim,Test}}$ ; 2) a weighted average of the 2 million squared differences was computed using a Gaussian kernel centered at $f_{\mathrm{resp}}^{\prime }$ and *ϵ*′ with the standard deviation of 0.015 Hz and 0.075 for the *f*_*resp*_ and *ϵ*, respectively; 3) the square root of the weighted average resulted in $\sigma \left(f_{\mathrm{resp}}^{\prime },\epsilon^{\prime }\right)$ .

In the present study, all versions of the CNN were trained and tested using 1-min segments, with one exception: An additional CNN $C_{RR,Resp,AFR}^{2.5min}$ was trained and tested using $X^{*}=\left[X_{RR}^{\mathrm{Sim,2.5}};X_{\mathrm{Resp}}^{\mathrm{Sim,2.5}};X_{\mathrm{AFR}}^{\mathrm{Sim,2.5}}\right]$ containing 2.5-minute-long segments to investigate the impact of segment length on the RMSE. For $C_{RR,Resp,AFR}^{2.5min}$ , ten realizations were trained with additional unique training and validation datasets, $X^{*Sim,Train}$ and $X^{\mathrm{Sim},\mathrm{Val}}$ , respectively, containing 100,000 parameter sets each. Apart from the different segment lengths, the additional datasets were generated as described in Section 2.3.2.

#### 2.4.5 Estimation of respiratory modulation in clinical data

The CNN $C_{RR,Resp,AFR}$ was used for estimating *a*_*resp*_ in the clinical deep breathing test data, described in Section 2.1. The clinical estimates were used to investigate differences in ${\hat{a}}_{\mathrm{resp}}$ between deep breathing and normal breathing using Monte Carlo sampling. Using these samples, the probabilities of the following three scenarios were computed for each patient: 1) the highest ${\hat{a}}_{\mathrm{resp}}$ was achieved for deep breathing, 2) the lowest ${\hat{a}}_{\mathrm{resp}}$ was achieved for deep breathing and 3) the highest and lowest ${\hat{a}}_{\mathrm{resp}}$ did not correspond to deep breathing. To draw the samples for each 1-min segment in $X^{\mathrm{Clin,Test}}$ , the estimate ${\hat{a}}_{\mathrm{resp}}$ was determined using the CNN $C_{RR,Resp,AFR}$ , while the $f_{\mathrm{resp}}^{\prime }$ and *ϵ*′ were estimated by the ${\hat{f}}_{\mathrm{resp}}$ and *ϵ*(**w**, *τ*, **Z**) described in Section 2.2.4. Next, values of ${\hat{a}}_{\mathrm{resp}}$ were resampled 100,000 times for each 1-min segment in $S_{\mathrm{seg}}^{*}$ . The samples were drawn from Gaussian distributions with ${\hat{a}}_{\mathrm{resp}}$ as mean and $\sigma \left(f_{\mathrm{resp}}^{\prime },\epsilon^{\prime }\right)$ described in Section 2.4.4 as standard deviation.

## 3 Results

### 3.1 Analysis of clinical data

**FIGURE 3:**
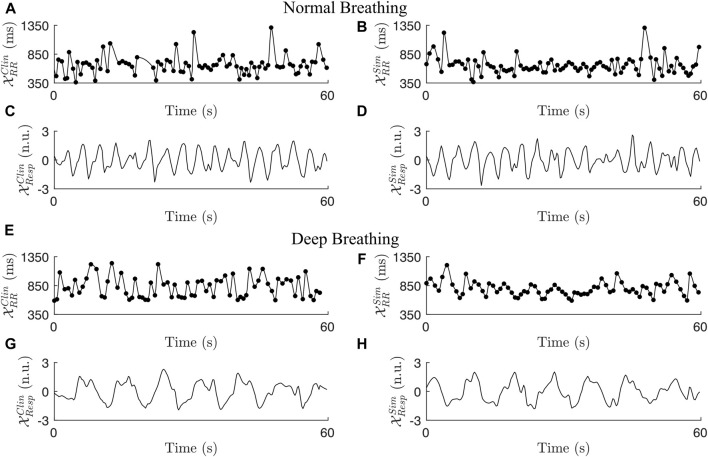
Two examples of clinical RR series (A + E), simulated RR series (B + F), clinical respiration signals (C + G), and simulated respiration signals (D + H) during normal breathing **(A–D)** and deep breathing **(E–H)**.

**FIGURE 4:**
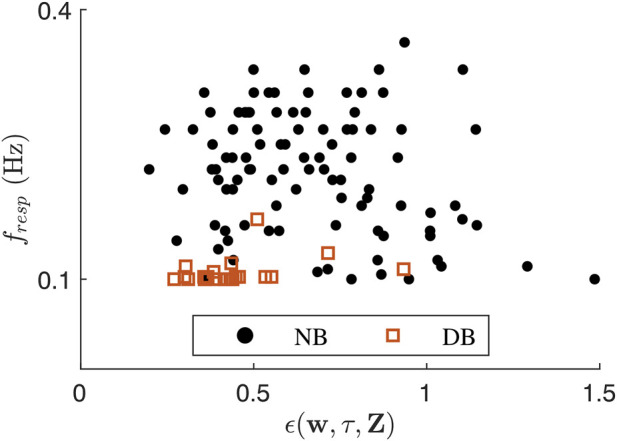
Scatter plot showing *ϵ*(**w**, *τ*, **Z**) over ${\hat{f}}_{\mathrm{resp}}$ for each 1-min segment during normal breathing (NB) and deep breathing (DB).

The length of the interpolated RR series varied between patients depending on the duration of the recordings; during normal breathing, the length of the RR series was in the range between 288 s and 328 s; during deep breathing, the length of the RR series was in the range between 57 s and 72 s. Statistics quantifying the clinical dataset are shown in Table 3. In accordance with the exclusion criteria defined in Section 2.2.5, 98 out of 120 non-overlapping 1-min segments remained in the normal breathing data and 22 out of 28 1-min segments remained in the deep breathing data. Typical examples of a clinical ECG-derived RR series $X_{RR}^{\mathrm{Clin}}$ and joint-lead respiration signal $X_{\mathrm{Resp}}^{\mathrm{Clin}}$ during normal breathing and deep breathing, respectively, are shown in Figure 3. The characteristics of these signals, listed in Table 4 are within 1 standard deviation of the population mean (cf. Table 3). Fluctuations in the clinical RR series matching the respiration frequencies were not clearly visible and *F*_*RR*_(*f*_*resp*_)/*∑*_*f*_ *F*_*RR*_(*f*) was always below 7%. The respiration signals estimated from clinical data had *ϵ*(**w**, *τ*, **Z**) ranging between 0.198 and 1.485. The clinical value pairs of *ϵ*(**w**, *τ*, **Z**) and respiration frequency f^resp are shown in Figure 4. There was a statistically significant weak negative correlation between f^resp and *ϵ*(**w**, *τ*, **Z**) in the clinical data during normal breathing (*r* = −.217, *p* = 0.032), but no significant correlation during deep breathing.

**TABLE 3:** Characteristics of clinical and simulated data.

	Clinical data $X^{\mathrm{Clin,Test}}$		Simulated data	
	Normal breathing	Deep breathing	Training Data	Testing Data
			[ $X^{\mathrm{Sim,Train}}{;X}^{\mathrm{Sim},\mathrm{Val}}$ ]	$X^{\mathrm{Sim,Test}}$
Number of X	98	22	10 ⋅ 2 ⋅ 100,000	2,000,000
RR mean (ms)	763 ± 173	747 ± 162	676 ± 164^†^	676 ± 164^†^
RR RMSSD (ms)	262 ± 100	230 ± 60	188 ± 60^†,‡^	185 ± 58^†,‡^
RR sample entropy	2.08 ± 0.49	2.18 ± 0.63	1.53 ± 0.39^†,‡^	1.52 ± 0.38^†,‡^
*F*_*RR*_(*f*_*resp*_)/*∑*_*f*_ *F*_*RR*_(*f*)(%)	2.5 ± 1.3	1.1 ± 0.8	3.4 ± 1.8^†,‡^	3.3 ± 1.7^†,‡^
$\bar{AFR}$ (Hz)	6.99 ± 0.7	6.95 ± 0.71	5.97 ± 1.57^†,‡^	5.96 ± 1.57^†,‡^
*f*_*resp*_(Hz)	0.220 ± 0.067	0.107 ± 0.015	0.263 ± 0.085^†,‡^	0.261 ± 0.085^†,‡^
*ϵ*	0.66 ± 0.25	0.44 ± 0.15	0.64 ± 0.27^‡^	0.64 ± 0.27^‡^
*a*_*resp*_	0.282 ± 0.101	0.285 ± 0.131	0.200 ± 0.173^†,‡^	0.200 ± 0.115^†,‡^

^†^ *p*< 0.05 vs. normal breathing. ^‡^ *p*< 0.05 vs. deep breathing. The training data is divided into 20 datasets with equal size to train the 10 realizations of the CNN with unique $X^{\mathrm{Sim,Train}}$ and $X^{\mathrm{Sim},\mathrm{Val}}$ . The variables $\bar{AFR}$ , *f*_*resp*_, and *a*_*resp*_ characterize estimates in the clinical data and model parameters in the simulated data.

**TABLE 4:** Characteristics of the clinical and simulated examples shown in Figure 3.

Signals	RR mean (ms)	RR RMSSD (ms)	RR sample entropy	*a*_*resp*_	*f*_*resp*_ (Hz)	*η*	*ϵ*(w, *τ*, Z)
A/C	661	250	1.85	-	0.286	-	0.47
B/D	651	204	1.91	0.36	0.288	2.48	0.76
E/G	818	251	2.28	-	0.118	-	0.44
F/H	792	138	1.97	0.05	0.116	1.46	0.45

### 3.2 Simulated RR series and respiration signals

The statistics quantifying $X^{\mathrm{Sim,Train}}$ , $X^{\mathrm{Sim},\mathrm{Val}}$ and $X^{\mathrm{Sim,Test}}$ are shown in Table 3 together with $X^{\mathrm{Clin,Test}}$ . The simulated datasets were created according to the description in Section 2.3 and compared to the clinical data using the unpaired *t*-test. It should be noted that although there are significant differences between the characteristics of the clinical and simulated data, the distributions of the simulated data cover the distribution of the clinical data. The heart rate was on average slightly faster and more regular in $X^{\mathrm{Sim}}$ than in $X^{\mathrm{Clin}}$ , as indicated by the differences in RR mean, RR RMSSD, and RR sample entropy. Further, the RR series in $X^{\mathrm{Sim}}$ showed on average more visible fluctuations matching the respiration frequency compared to the RR series in $X^{\mathrm{Clin}}$ , as indicated by the difference in *F*_*RR*_(*f*_*resp*_)/*∑*_*f*_ *F*_*RR*_(*f*). The $\bar{AFR}$ was on average slightly lower in $X^{\mathrm{Sim}}$ than in $X^{\mathrm{Clin}}$ , whereas *f*_*resp*_ was slightly higher. In normal breathing, *ϵ* in $X^{\mathrm{Clin}}$ was comparable to $X^{\mathrm{Sim}}$ ; however, in deep breathing, *ϵ* was lower in $X^{\mathrm{Clin}}$ than in $X^{\mathrm{Sim}}$ .

Examples of a simulated RR series $X_{RR}^{\mathrm{Sim}}$ and joint-lead respiration signal $X_{\mathrm{Resp}}^{\mathrm{Sim}}$ resembling clinical signals during normal breathing and deep breathing, respectively, are shown in Figure 3. The signals were chosen based on similarities to the clinical ECG-derived signals in the RR series characteristics and respiration signal morphology. The characteristics of these signals are listed in Table 4. Note, that while the peak-to-peak amplitude of respiration-induced autonomic modulation *a*_*resp*_ is high during normal breathing and low during deep breathing in this example, a general conclusion about the *a*_*resp*_ values of the clinical signals can not be drawn from this comparison and is not intended. When emulating lead-specific EDR signals and adding Gaussian noise with standard deviation *η*, the simulated data showed a strong correlation between *η* and *ϵ* (*r* = 0.89, *p* < 10^–5^). The examples in Figure 3 are representative of this correlation with the *η* and *ϵ* listed in Table 4, where $X_{\mathrm{Resp}}^{\mathrm{Sim}}$ in Figure 3D was generated with a higher *η* and showed a higher *ϵ* compared to $X_{\mathrm{Resp}}^{\mathrm{Sim}}$ in Figure 3H.

### 3.3 Accuracy of convolutional neural network

**FIGURE 5:**
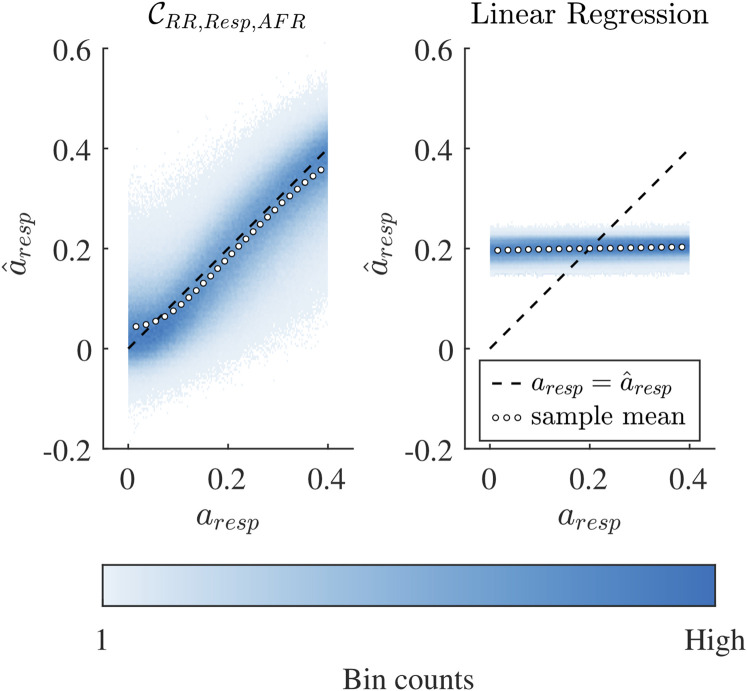
Binned scatter plot of estimated ${\hat{a}}_{\mathrm{resp}}$ versus true *a*_*resp*_ for the CNN $C_{RR,Resp,AFR}$ and linear regression $L_{RR,Resp,AFR}$ , where both were based on the same input data $X=\left[X_{RR}^{\mathrm{Sim}};X_{\mathrm{Resp}}^{\mathrm{Sim}};X_{\mathrm{AFR}}^{\mathrm{Sim}}\right]$ . The black dotted line shows where ${\hat{a}}_{\mathrm{resp}}$ is equal to *a*_*resp*_. The white dotted line shows the sample mean of the ${\hat{a}}_{\mathrm{resp}}$ estimation.

All CNNs $C_{RR}$ , $C_{\mathrm{Resp}}$ , $C_{\mathrm{AFR}}$ , $C_{RR,Resp}$ , $C_{RR,AFR}$ , $C_{\mathrm{Resp,AFR}}$ and $C_{RR,Resp,AFR}$ , described in Section 2.4.2 and trained according to Section 2.4.3, were tested using $X^{\mathrm{Sim,Test}}$ described in Section 2.3.2. The resulting distribution of estimated ${\hat{a}}_{\mathrm{resp}}$ over true *a*_*resp*_ for $C_{RR,Resp,AFR}$ is shown in Figure 5. Also displayed in Figure 5 for comparison is the corresponding distribution for estimation using linear regression $L_{RR,Resp,AFR}$ based on the same data $X=\left[X_{RR}^{\mathrm{Sim}};X_{\mathrm{Resp}}^{\mathrm{Sim}};X_{\mathrm{AFR}}^{\mathrm{Sim}}\right]$ . The RMSE, Pearson sample correlation and *R* ^2^ are listed for the seven CNN versions and $L_{RR,Resp,AFR}$ in Table 5. The $C_{RR,Resp,AFR}$ resulted in the lowest RMSE and highest correlation and *R* ^2^. The CNNs $C_{\mathrm{AFR}}$ , $C_{\mathrm{Resp}}$ and $C_{\mathrm{Resp,AFR}}$ without RR series in the input data performed poorly. The $C_{RR}$ estimated ${\hat{a}}_{\mathrm{resp}}$ with an RMSE of 0.074, where the addition of $X_{\mathrm{Resp}}^{\mathrm{Sim}}$ or $X_{\mathrm{AFR}}^{\mathrm{Sim}}$ to the input improved the accuracy of the ${\hat{a}}_{\mathrm{resp}}$ estimation slightly.

**TABLE 5:** RMSE, Pearson sample correlation and *R* ^2^ of the seven CNN versions and linear regression $L_{RR,Resp,AFR}$ using 1-min segments, and $C_{RR,Resp,AFR}^{2.5min}$ using 2.5-min segments.

	RMSE	Pearson correlation *r*	*R* ^2^
$C_{RR,Resp,AFR}^{2.5min}$	0.050	0.923	0.816
$C_{RR,Resp,AFR}$	0.066	0.855	0.674
$C_{RR,Resp}$	0.070	0.830	0.636
$C_{RR,AFR}$	0.070	0.837	0.630
$C_{RR}$	0.074	0.805	0.585
$C_{\mathrm{Resp,AFR}}$	0.098	0.583	0.284
$C_{\mathrm{Resp}}$	0.101	0.513	0.231
$C_{\mathrm{AFR}}$	0.115	0.073	0.001
$L_{RR,Resp,AFR}$	0.119	0.037	−0.068

**FIGURE 6:**
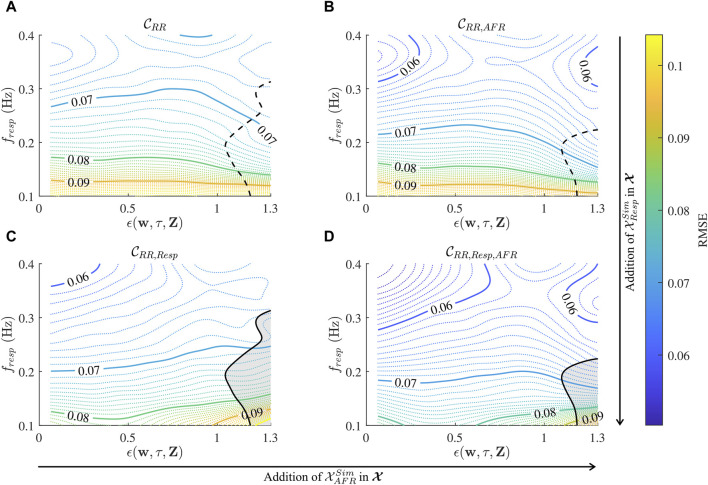
Contour plot of local RMSE estimates over a range of $f_{\mathrm{resp}}^{\prime }$ and *ϵ* ^′^ for $C_{RR}$ **(A)**, $C_{RR,AFR}$ **(B)**, $C_{RR,Resp}$ **(C)** and $C_{RR,Resp,AFR}$ **(D)**. Except for the grey region, the CNNs in **(C, D)** have a higher accuracy than the CNNs in **(A,B)** respectively.

For $C_{RR}$ , $C_{RR,AFR}$ , $C_{RR,Resp}$ and $C_{RR,Resp,AFR}$ , the local RMSE of ${\hat{a}}_{\mathrm{resp}}$ for specific $f_{\mathrm{resp}}^{\prime }$ and *ϵ*′ were computed according to Section 2.4.4 and illustrated in Figure 6. It can be seen in all four contour plots that the RMSE is dependent on $f_{\mathrm{resp}}^{\prime }$ and *ϵ*′. The CNNs produce more accurate estimations for data with a high $f_{\mathrm{resp}}^{\prime }$ and low *ϵ*′, however, the RMSE is more sensitive to changes in $f_{\mathrm{resp}}^{\prime }$ . Adding $X_{\mathrm{AFR}}^{\mathrm{Sim}}$ to the input improves the RMSE for all values of *f*_*resp*_ and *ϵ*. While the addition of $X_{\mathrm{Resp}}^{\mathrm{Sim}}$ to the input improves the RMSE for most $f_{\mathrm{resp}}^{\prime }$ and *ϵ*′, it worsens the RMSE for high *ϵ*′ and low $f_{\mathrm{resp}}^{\prime }$ as indicated in Figure 6. Within the indicated region, the accuracy of ${\hat{a}}_{\mathrm{resp}}$ is higher without $X_{\mathrm{Resp}}^{\mathrm{Sim}}$ in the input data.

The accuracy of the CNN improves with longer input data, indicated by the fact that the RMSE of $C_{RR,Resp,AFR}^{2.5min}$ was 0.050. The RMSE, Pearson sample correlation and *R* ^2^ is listed for $C_{RR,Resp,AFR}^{2.5min}$ in Table 5. The RMSE improved for all values of *ϵ*′ and $f_{\mathrm{resp}}^{\prime }$ , whereas the local RMSE improved especially at lower $f_{\mathrm{resp}}^{\prime }$ (data not shown).

### 3.4 Estimation of respiration-induced autonomic modulation in clinical data

**FIGURE 7:**
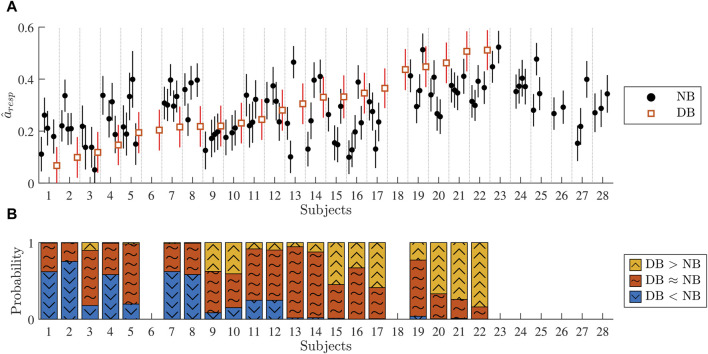
**(A)** Black dots correspond to the estimated ${\hat{a}}_{\mathrm{resp}}$ of 1-min segments during normal breathing (NB) and red squares correspond to ${\hat{a}}_{\mathrm{resp}}$ of 1-min segments during deep breathing (DB). The vertical lines correspond to ± *σ*(*f*_*resp*_, *ϵ*), where the local RMSE *σ*(*f*_*resp*_, *ϵ*) is taken from Figure 6D. **(B)** Probabilities of *a*_*resp*_ being higher in DB than in NB (yellow, arrow-up), similar in DB and NB (red, tilde), and lower in DB than in NB (blue, arrow-down).

The CNN $C_{RR,Resp,AFR}$ was used to obtain ${\hat{a}}_{\mathrm{resp}}$ from the clinical ECG-derived features in $X=\left[X_{RR}^{\mathrm{Clin}};X_{\mathrm{Resp}}^{\mathrm{Clin}};X_{\mathrm{AFR}}^{\mathrm{Clin}}\right]$ . The resulting ${\hat{a}}_{\mathrm{resp}}$ for 1-min segments during normal breathing and deep breathing are shown in Figure 7. There was high interpatient variability in ${\hat{a}}_{\mathrm{resp}}$ in the study population and no clear relation was found between ${\hat{a}}_{\mathrm{resp}}$ during normal breathing and deep breathing. No significant correlation was found between a change in respiration frequency f^respDB−f^respNB and a change in respiration-induced autonomic modulation ${\hat{a}}_{\mathrm{resp}}^{DB}-{\hat{a}}_{\mathrm{resp}}^{NB}$ .

The vertical lines around ${\hat{a}}_{\mathrm{resp}}$ in Figure 7A correspond to ± *σ*(*f*_*resp*_, *ϵ*), described in Section 2.4.4 and is used for the Monte Carlo sampling described in Section 2.4.5. For 20 subjects, ${\hat{a}}_{\mathrm{resp}}$ was available for at least one segment during normal breathing and one segment during deep breathing (cf. exclusion criteria in Section 2.2.5). For those 20 subjects, Monte Carlo sampling was used to investigate whether ${\hat{a}}_{\mathrm{resp}}$ is larger during deep breathing than during normal breathing as described in Section 2.4.5. As illustrated in Figure 7B: it was most likely for 5 patients that the highest *a*_*resp*_ was achieved for deep breathing; it was most likely for 5 patients that the lowest *a*_*resp*_ was achieved for deep breathing; and it was most likely for 10 patients that neither the highest nor lowest *a*_*resp*_ corresponded to deep breathing.

## 4 Discussion

To address the need for assessing autonomic dysfunction in patients with persistent AF, we developed a method to extract respiration-induced autonomic modulation in the AV node conduction properties from ECG data in AF. We focused on respiration-induced autonomic modulation because respiration is always present, respiration can be extracted from ECG signals, and abnormal respiration-induced autonomic modulation is often an early sign of autonomic dysfunction (Bernardi et al., 2001). To achieve this we extended our AV node model (Plappert et al., 2022) to account for respiration-induced autonomic modulation by including a time-varying scaling factor in the formulations of the AV nodal refractory period and conduction delay. We trained a 1D-CNN on simulated 1-min segments of RR series, respiration signals, and mean arrival rate of atrial impulses which replicate clinical data to estimate the peak-to-peak amplitude of respiration-induced autonomic modulation *a*_*resp*_. We evaluated the network on simulated data and the results indicated that *a*_*resp*_ can be estimated with an RMSE of 0.066, corresponding to a sixth of the expected range for *a*_*resp*_ between 0 and 0.4. Previous studies indicate that AF progression is linked to impaired baroreflex sensitivity (van den Berg et al., 2001; Field et al., 2016; Miyoshi et al., 2020; Ferreira et al., 2023; Wang et al., 2023). Additionally, in healthy subjects, the baroreflex is a major contributor to respiration-induced autonomic modulation (Piepoli et al., 1997). Together, this suggests that our proposed estimate for respiration-induced autonomic modulation, *a*_*resp*_, holds potential as a marker for AF progression. However, further studies are needed to confirm this relationship.

Initial results from analysis of clinical ECG data from patients in AF (cf. Figure 7A) indicate that during normal breathing, ${\hat{a}}_{\mathrm{resp}}$ is often consistent between consecutive 1-min segments from the same patient, and displays a systematic difference between patients, suggesting that ${\hat{a}}_{\mathrm{resp}}$ is reproducible and sensitive. During controlled breathing at 0.1 Hz, ${\hat{a}}_{\mathrm{resp}}$ displayed a large interpatient variability (cf. Figure 7A) and represented the most extreme value in 10 of 20 patients (cf. Figure 7B), further supporting an adequate sensitivity. However, further studies with a larger study population and repeated tests with multiple fixed respiration rates are needed to verify reproducibility and sensitivity. The respiration rate of 0.1 Hz is associated with a maximized HRV response and baroreflex sensitivity in NSR (Russo et al., 2017), and hence we expected an increase in ${\hat{a}}_{\mathrm{resp}}$ during deep breathing. However, results from the Monte Carlo sampling showed that ${\hat{a}}_{\mathrm{resp}}$ increased in response to deep breathing in 5 patients, decreased in 5 patients, and remained the same in 10 patients. There are several possible reasons for this, e.g., the differences in respiration rate during normal breathing (cf. Figure 4), individual variation in the cardiorespiratory system resonance frequency (Russo et al., 2017), and differences in autonomic remodeling. It should be noted that the individual differences cannot be entirely attributed to the differences in respiration rates, since there was no correlation between changes in respiration rate and changes in *a*_*resp*_. Due to the small patient group and lack of ground truth data in this study, future work with access to ground truth data is required to investigate if there is a correlation between *a*_*resp*_ and baroreflex sensitivity, and whether *a*_*resp*_ is a diagnostic marker for autonomic dysfunction. A re-evaluation of 50% of the SCAPIS population is currently underway within the SCAPIS2 study, and the data could allow for the investigation of whether the *a*_*resp*_ estimates decrease over time in the same AF patients, which would indicate a progression in autonomic remodeling. Furthermore, the collected data could be used for phenotyping the relation between respiration-induced autonomic modulation, autonomic dysfunction, and AF progression.

In our previous model formulation, we accounted for the autonomic modulation by introducing constant scaling factors for the refractory period and conduction delay (Plappert et al., 2022). With the scaling of AV nodal conduction properties, it was shown that the incorporation of ANS-induced changes in the model allowed better replication of several statistical properties of clinical RR series obtained from tilt tests. In the present study, this approach was further developed by using a time-varying scaling factor *A* ^*P*^(*t*) to account for respiration-induced autonomic modulation in AV nodal conduction properties based on the assumption that some degree of respiration-induced autonomic modulation generally influences RR series characteristics during AF. We model respiration-induced autonomic modulation as a joint increase in AV nodal refractoriness and conduction delay in response to exhalation and a joint decrease in AV nodal refractoriness and conduction delay in response to inhalation. It is known that respiration modulates the parasympathetic activity, where inspiration decreases vagal activity and expiration increases vagal activity (Katona et al., 1970; Russo et al., 2017). Many electrophysiological (EP) studies have demonstrated that an increase in parasympathetic activity causes an increase in AV nodal conduction delay; studies in dogs reported an increased conduction delay with vagal stimulation (Irisawa et al., 1971; Spear and Moore, 1973; Martin, 1975; Nayebpour et al., 1990; Pirola and Potter, 1990; Goldberger et al., 1999) and acetylcholine administration (Priola et al., 1983). Furthermore, an increase in parasympathetic activity with vagal stimulation in dogs has been demonstrated to increase the AV nodal refractory period (Spear and Moore, 1973; Nayebpour et al., 1990; Goldberger et al., 1999). For a decrease in parasympathetic activity with atropine, EP studies demonstrate that the AV nodal conduction delay decreases in dogs (Irisawa et al., 1971) and humans (Lister et al., 1965; Akhtar et al., 1974), and the AV nodal refractory period also decreases in humans (Akhtar et al., 1974).

The assumption that some degree of respiration-induced autonomic modulation generally influences the RR series characteristics during AF is also indicated by the fact that some AF patients display clear fluctuations in their RR series matching their respiration frequency (Rawles et al., 1989; Chandler and Trewby, 1994; Nagayoshi et al., 1997). Such fluctuations could also be seen in simulated RR series for some AV node model parameter sets. During model development, we noticed that an increase in *a*_*resp*_ leads to an increase in the relative contribution of the respiration frequency in the frequency spectrum of the RR series with zero-mean *F*_*RR*_(*f*_*resp*_)/*∑*_*f*_ *F*_*RR*_(*f*) and an increase in the sample entropy of the RR series. We also noticed that an increase in *f*_*resp*_ leads to a decrease in *F*_*RR*_(*f*_*resp*_)/*∑*_*f*_ *F*_*RR*_(*f*) and an increase in the sample entropy of the RR series. When averaging over several realizations of RR series (data not shown), *F*_*RR*_(*f*_*resp*_)/*∑*_*f*_ *F*_*RR*_(*f*) could be clearly seen for most of the parameter sets but is usually masked in individual RR series segments by the irregularity of the RR series. Using cross-spectral analysis, no simple linear relationship has been found between respiration signal and RR series in AF patients, but a linear relationship was shown in NSR (Pitzalis et al., 1999). A possible reason for this is that the relationship between the RR series and respiration-induced autonomic modulation in AV nodal conduction properties during AF is complex and non-linear, emphasizing the need for a model-based approach. Besides some indications of fluctuations in the RR series, for most of the patients reported in (Rawles et al., 1989; Chandler and Trewby, 1994; Nagayoshi et al., 1997; Pitzalis et al., 1999; Pacchia et al., 2011) and also for the clinical data used in this study, no fluctuations in the RR series matching their respiration frequency were found. To match *F*_*RR*_(*f*_*resp*_)/*∑*_*f*_ *F*_*RR*_(*f*) in the clinical data which was always below 7%, parameter sets with a higher relative peak spectral energy were excluded from the simulated data (criterion 5 in Section 2.3.2). The RR series characteristics of the simulated data differed significantly from both the normal breathing and deep breathing data (cf. Table 3). Simulated data with RR series characteristics more similar to the clinical data could be generated by imposing stricter exclusion criteria, e.g., increasing the lower bounds for irregularity and variability set by criteria 3 and 4 in Section 2.3.2. However, the simulated data still included signals resembling the clinical data, and the wider range of characteristics likely improved the CNN training by facilitating generalization across a broader range of RR-series. Nevertheless, it is assumed that by the sheer size of the simulated datasets and the conservative model parameter ranges, there will be simulated RR series in the dataset that resemble the clinical data.

The lead-specific respiration signals were computed using the slope range method which was designed for ECG data during AF (Kontaxis et al., 2020) and found to be one of the best performing and simplest methods for lead-specific respiration signal extraction (Varon et al., 2020). The result of the lead-specific respiration signal extraction can be improved when combining respiration signals from multiple ECG leads with a joint-lead respiration signal. Previously, the principal component analysis (PCA) has been used to extract joint-lead respiration signals from the clinical data used in this study (Abdollahpur et al., 2022). However, the principal components were sensitive to high variance noise as the PCA is based on second-order statistics. To address this issue, we developed a novel approach for robust fusion of lead-specific respiration signals based on the *π*CA (Sameni et al., 2008). Under the assumption that the respiration signal has a periodic structure where the respiration frequency and volume between breaths are constant, the *π*CA is more suitable for the extraction of joint-lead respiration signals compared to other blind-source separation methods, such as the PCA and basic independent component analysis (ICA). This is because the *π*CA finds the linear mixture of lead-specific respiration signals with maximal periodic structure, whereas the PCA and basic ICA are based on second-order and fourth-order statistics, respectively. We assume that the respiration frequency and volume between breaths do not vary much in 1-min segments, making the *π*CA a suitable approach for the extraction of short joint-lead respiration signals. However, considering that the CNN $C_{RR,Resp,AFR}^{2.5min}$ performs better when using 2.5-min segments instead of 1-min segments, another method may be required for the extraction of longer joint-lead respiration signals.

The comparison between the CNN $C_{RR,Resp,AFR}$ and the linear regression $L_{RR,Resp,AFR}$ shown in Figure 5 demonstrates that the relation between the ECG-derived features, i.e., RR series, respiration signal and mean atrial arrival rate to *a*_*resp*_ is complex and nonlinear. The $L_{RR,Resp,AFR}$ was unable to estimate *a*_*resp*_ (Pearson sample correlation *r* = 0.037) and performed clearly worse in estimating *a*_*resp*_ than the investigated CNN $C_{RR,Resp,AFR}$ (*r* = 0.855). It should be noted that the purpose of this comparison is to exclude the possibility that there is a simple and linear relationship between the ECG-derived features and *a*_*resp*_. We also investigated the performance of a Gaussian kernel support vector machine for estimating *a*_*resp*_, representing a classical non-linear algorithm. Results were slightly better than for the linear model (*r* = 0.254, details in Supplementary Section 1), but still clearly worse than for the CNN. The advantage of the CNN over the less flexible models might be partially due to its ability to implicitly extract more complex features from the respiration signal and RR series in the early layers. While no such set of features is currently known for this problem, this leads us to speculate that some type of additional, pre-defined feature extraction step might improve the performance of also the simpler models. However, this task is far from trivial and lies outside the scope of the present study, but may nevertheless offer an interesting avenue for future work, e.g., by investigating statistical properties of the RR series based on RMS of successive RR interval differences or entropy measures.

In this study, we only investigate the performance of one basic CNN architecture. While some variations on this were tested during the neural network development, no extensive investigation has been performed and there is always the possibility that alternative architectures or algorithms may further increase the performance for the present task. For instance, a recent study suggests that combining the regression loss with a classification loss during training might improve regression results on imbalanced data (Pintea et al., 2023). The CNN described in this study requires the RR series for the estimation of *a*_*resp*_ and the mean atrial arrival rate always improved the estimation. In this evaluation, however, *μ* was set to the correct value; we did not account for estimation errors that are most likely present in real data since AFR provides a crude estimate of the atrial arrival rate. Moreover, the addition of the respiration signal only improves the estimation when of sufficient quality as quantified by *ϵ*. The linear dependence between *η* and *ϵ* supports our assumption of *ϵ* as a marker of respiration signal quality (cf. Section 3.2). Whereas the addition of the respiration signal and mean atrial arrival rate can improve the estimation of ${\hat{a}}_{\mathrm{resp}}$ , a method based on RR series only is less sensitive to noise in the recordings. Potentially, the RR series could be extracted from pulse watch data, allowing for continuous monitoring of *a*_*resp*_ in a wide range of applications.

The performance of the CNN is dependent on *f*_*resp*_ and *ϵ* (cf. Figure 6), where *f*_*resp*_ appears to have a larger impact on the performance than *ϵ*. The marker of respiration signal quality *ϵ* was not used as an exclusion criterion for 1-min segments, because the addition of $X_{\mathrm{Resp}}^{\mathrm{Sim}}$ to the input only slightly improved the accuracy of the ${\hat{a}}_{\mathrm{resp}}$ estimation and the influence of *ϵ* on the RMSE compared to *f*_*resp*_ was small. Instead, *ϵ* was used to choose the best combination of non-overlapping 1-min segments. Interestingly, the performance of the CNNs $C_{RR}$ , $C_{\mathrm{AFR}}$ , $C_{RR,AFR}$ still show a slight dependence on *ϵ* although this parameter quantifies the non-periodicity and signal quality of $X_{\mathrm{Resp}}^{\mathrm{Sim}}$ (cf. Figure 6). This suggests that *ϵ* carries information about the RR interval series, and may indicate that the distribution of AV node model parameters varies over different *ϵ* and that different subsets of model parameters result in different local RMSEs. One possible explanation why the impact of *f*_*resp*_ on the performance is prominent may be that there are fewer respiratory cycles in the 1-min segment at lower *f*_*resp*_. When using 2.5-min segments in the input data, the performance of the CNN $C_{RR,Resp,AFR}^{2.5min}$ improved overall, especially at lower *f*_*resp*_. The segment length was set to 1 min in this study due to the recording length of 1 min during deep breathing.

There are several limitations of the present study. We assume for simplicity that the variations in AV nodal refractoriness are similar to the variations in AV nodal conduction delay. We also assume that the variations in AV nodal refractoriness and conduction delay are similar between SP and FP. Moreover, the model does not include phase shifts between the RR series and respiration signal for different respiration frequencies (Angelone and Coulter, 1964), or effects of respiration volume (Grossman and Taylor, 2007). Hence, a different scaling for the refractory period and conduction delay, a different scaling for the SP and FP, a phase shift between the RR series and respiration signal, and an inclusion of respiration volume might form interesting directions for future model improvements. We did not account for respiration-induced modulation in the AA series, because the modulation is small during AF (Celotto et al., 2020; Abdollahpur et al., 2022). When choosing the bounded uniform distribution of *a*_*resp*_ for the training and testing dataset, we made a tradeoff between bias and variance. The reason why *a*_*resp*_ was randomly drawn from U[−0.1,0.5] in the training data and randomly drawn from U[0,0.4] in the testing data of the CNN is to reduce the bias in the ${\hat{a}}_{\mathrm{resp}}$ estimation (cf. Figure 5). Without extending the range of *a*_*resp*_ in the training data, the sample mean of the ${\hat{a}}_{\mathrm{resp}}$ diverged more from *a*_*resp*_ at values close to 0 and 0.4. However, the accuracy of the CNNs decreased by extending the range of *a*_*resp*_ in the training data. While plenty of simulated data with *a*_*resp*_ ground truth can be generated using the AV node model, there was no *a*_*resp*_ ground truth available for the clinical dataset and its size was limited. A viable approach to obtain *a*_*resp*_ ground truth may be through measurements of vagal nerve activity, which were previously collected in a large number of experimental studies, e.g., to assess the relationship to HRV during sinus rhythm in rat models (Marmerstein et al., 2021) and to assess the relationship to paroxysmal AF episodes in canine models (Tan et al., 2008). Furthermore, ultrasound-guided microneurography was proposed to obtain *in vivo* recordings from the human vagus nerve (Ottaviani et al., 2020) and results from analysis of intraneural recordings from cervical nerve in awake humans suggest that cardiac and respiration-induced autonomic modulation during normal sinus rhythm can be identified (Patros et al., 2022). Another possibility would be indirect quantification of respiration-induced autonomic modulation via acetylcholine concentration (Świt et al., 2023), but we are not aware of any procedure or method that would produce the required time resolution.

## 5 Conclusion

We presented an extended AV node model that accounts for respiration-induced autonomic modulation in conduction delay and refractory period. We trained a 1D-CNN to estimate the respiration-induced autonomic modulation in the AV node with simulated RR series, respiration signal, and mean atrial arrival rate which replicates clinical ECG-derived data. Using simulated data, we demonstrated that the respiration-induced autonomic modulation can be estimated using the 1D-CNN from RR series alone and that the estimation is improved when adding a respiration signal and AFR. Initial results from analysis of ECG data from 20 patients performing a deep breathing task suggest that our proposed estimate of respiration-induced autonomic modulation *a*_*resp*_, is reproducible and sufficiently sensitive to monitor changes and to detect individual differences. A reduced estimate of *a*_*resp*_ may possibly indicate some degree of autonomic dysfunction. However, further studies are needed to verify the reproducibility, sensitivity, and clinical significance of *a*_*resp*_.

## Data Availability

The datasets presented in this article are not readily available because they are owned by SCAPIS. Requests to access the datasets should be directed to info@scapis.org (www.scapis.org/data-access/). The code for the model together with a user example can be found at https://github.com/PlappertF/ECG-based_estimation_of_respiration-induced_autonomic_modulation_of_AV_nodal_conduction_during_AF.

## References

[B1] Abdollahpur M. Engström G. Platonov P. G. Sandberg F. (2022). A subspace projection approach to quantify respiratory variations in the f-wave frequency trend. Front. Physiol. 13, 976925. 10.3389/fphys.2022.976925 36200057 PMC9527347

[B2] Akhtar M. Damato A. N. Caracta A. R. Batsford W. P. Josephson M. E. Lau S. H. (1974). Electrophysiologic effects of atropine on atrioventricular conduction studied by his bundle electrogram. Am. J. Cardiol. 33, 333–343. 10.1016/0002-9149(74)90313-0 4812554

[B3] Angelone A. Coulter N. A. (1964). Respiratory sinus arrhythmia: a frequency dependent phenomenon. J. Appl. Physiol. 19, 479–482. 10.1152/jappl.1964.19.3.479 14173545

[B4] Bergström G. Berglund G. Blomberg A. Brandberg J. Engström G. Engvall J. (2015). The Swedish CArdioPulmonary BioImage Study: objectives and design. J. Intern. Med. 278, 645–659. 10.1111/joim.12384 26096600 PMC4744991

[B5] Bernardi L. Porta C. Gabutti A. Spicuzza L. Sleight P. (2001). Modulatory effects of respiration. Auton. Neurosci. 90, 47–56. 10.1016/S1566-0702(01)00267-3 11485292

[B6] Billette J. Tadros R. (2019). An integrated overview of AV node physiology. Pacing Clin. Electrophysiol. 42, 805–820. 10.1111/pace.13734 31144331

[B7] Celotto C. Sánchez C. Mountris K. A. Abdollahpur M. Sandberg F. Laguna P. (2020). Relationship between atrial oscillatory acetylcholine release pattern and f-wave frequency modulation: a computational and experimental study. Comput. Cardiol., 1–4. 10.22489/CinC.2020.303

[B8] Chandler S. T. Trewby P. N. (1994). Is respiratory sinus arrhythmia present in atrial fibrillation? a study using two quantitative methods. Med. Eng. Phys. 16, 334–337. 10.1016/1350-4533(94)90061-2 7952670

[B9] Climent A. M. Atienza F. Millet J. Guillem M. S. (2011a). Generation of realistic atrial to atrial interval series during atrial fibrillation. Med. Biol. Eng. Comput. 49, 1261–1268. 10.1007/s11517-011-0823-2 21830052

[B10] Climent A. M. Guillem M. S. Zhang Y. Millet J. Mazgalev T. N. (2011b). Functional mathematical model of dual pathway AV nodal conduction. Am. J. Physiol. Heart Circ. Physiol. 300, 1393–1401. 10.1152/ajpheart.01175.2010 21257912

[B11] Cohen R. J. Berger R. D. Dushane T. E. (1983). A quantitative model for the ventricular response during atrial fibrillation. IEEE Trans. Biomed. Eng. 30, 769–781. 10.1109/TBME.1983.325077 6662535

[B12] Doste R. Lozano M. Jimenez-Perez G. Mont L. Berruezo A. Penela D. (2022). Training machine learning models with synthetic data improves the prediction of ventricular origin in outflow tract ventricular arrhythmias. Front. Physiol. 12, 909372. 10.3389/fphys.2022.909372 PMC9412034 36035489

[B13] Engström G. Hamrefors V. Fedorowski A. Persson A. Johansson M. E. Ostenfeld E. (2022). Cardiovagal function measured by the deep breathing test: relationships with coronary atherosclerosis. J. Am. Heart Assoc. 11, e024053. 10.1161/JAHA.121.024053 35352566 PMC9075454

[B14] Ferreira M. Laranjo S. Cunha P. Geraldes V. Oliveira M. Rocha I. (2023). Orthostatic stress and baroreflex sensitivity: a window into autonomic dysfunction in lone paroxysmal atrial fibrillation. J. Clin. Med. 12, 5857. 10.3390/jcm12185857 37762798 PMC10532155

[B15] Field M. E. Wasmund S. L. Page R. L. Hamdan M. H. (2016). Restoring sinus rhythm improves baroreflex function in patients with persistent atrial fibrillation. J. Am. Hear. Assoc. 5, e002997. 10.1161/jaha.115.002997 PMC4802450 26908410

[B16] George S. A. Faye N. R. Murillo-Berlioz A. Lee K. B. Trachiotis G. D. Efimov I. R. (2017). At the atrioventricular crossroads: dual pathway electrophysiology in the atrioventricular node and its underlying heterogeneities. Arrhythm. Electrophysiol. Rev. 6, 179–185. 10.15420/aer.2017.30.1 29326832 PMC5739891

[B17] Gheorghita B. A. Itu L. M. Sharma P. Suciu C. Wetzl J. Geppert C. (2022). Improving robustness of automatic cardiac function quantification from cine magnetic resonance imaging using synthetic image data. Sci. Rep. 12, 2391. 10.1038/s41598-022-06315-3 35165324 PMC8844403

[B18] Goldberger J. J. Kadish A. H. Johnson D. Qi X. (1999). New technique for vagal nerve stimulation. J. Neurosci. Methods. 91, 109–114. 10.1016/S0165-0270(99)00085-0 10522829

[B19] Grossman P. Taylor E. W. (2007). Toward understanding respiratory sinus arrhythmia: relations to cardiac vagal tone, evolution and biobehavioral functions. Biol. Psychol. 74, 263–285. 10.1016/j.biopsycho.2005.11.014 17081672

[B20] Hanna P. Dacey M. J. Brennan J. Moss A. Robbins S. Achanta S. (2021). Innervation and neuronal control of the mammalian sinoatrial node a comprehensive atlas. Circ. Res. 128, 1279–1296. 10.1161/CIRCRESAHA.120.318458 33629877 PMC8284939

[B21] Henriksson M. Corino V. D. Sörnmo L. Sandberg F. (2016). A statistical atrioventricular node model accounting for pathway switching during atrial fibrillation. IEEE Trans. Biomed. Eng. 63, 1842–1849. 10.1109/TBME.2015.2503562 26625403

[B22] Henriksson M. Petrėnas A. Marozas V. Sandberg F. Sörnmo L. (2018). Model-based assessment of f-wave signal quality in patients with atrial fibrillation. IEEE Trans. Biomed. Eng. 65, 2600–2611. 10.1109/TBME.2018.2810508 29993509

[B23] Hindricks G. Potpara T. Dagres N. Arbelo E. Bax J. J. Blomström-Lundqvist C. (2020). 2020 ESC Guidelines for the diagnosis and management of atrial fibrillation developed in collaboration with the European Association for Cardio-Thoracic Surgery (EACTS): the Task Force for the diagnosis and management of atrial fibrillation of the European Society of Cardiology (ESC) Developed with the special contribution of the European Heart Rhythm Association (EHRA) of the ESC. Eur. Heart J. 42, 373–498. 10.1093/eurheartj/ehaa612 32860505

[B24] Inada S. Shibata N. Iwata M. Haraguchi R. Ashihara T. Ikeda T. (2017). Simulation of ventricular rate control during atrial fibrillation using ionic channel blockers. J. Arrhythm. 33, 302–309. 10.1016/j.joa.2016.12.002 28765761 PMC5529332

[B25] Irisawa H. Caldwell W. M. Wilson M. F. (1971). Neural regulation of atrioventricular conduction. Jpn. J. Physiol. 21, 15–25. 10.2170/jjphysiol.21.15 5317233

[B26] Joglar J. A. Chung M. K. Armbruster A. L. Benjamin E. J. Chyou J. Y. Cronin E. M. (2023). 2023 ACC/AHA/ACCP/HRS guideline for the diagnosis and management of atrial fibrillation A report of the American college of cardiology/American heart association joint committee on clinical practice guidelines. J. Am. Coll. Cardiol. 83, 109–279. 10.1016/j.jacc.2023.08.017 38043043 PMC11104284

[B27] Kaisti M. Laitala J. Wong D. Airola A. (2023). Domain randomization using synthetic electrocardiograms for training neural networks. Artif. Intell. Med. 143, 102583. 10.1016/j.artmed.2023.102583 37673557

[B28] Karlsson M. Sandberg F. Ulimoen S. R. Wallman M. (2021). Non-invasive characterization of human AV-Nodal conduction delay and refractory period during atrial fibrillation. Front. Physiol. 12, 728955. 10.3389/fphys.2021.728955 34777001 PMC8584495

[B29] Katona P. G. Poitras J. W. Barnett G. O. Terry B. S. (1970). Cardiac vagal efferent activity and heart period in the carotid sinus reflex. Am. J. Physiol. 218, 1030–1037. 10.1152/ajplegacy.1970.218.4.1030 5435400

[B30] Kontaxis S. Lázaro J. Corino V. D. Sandberg F. Bailón R. Laguna P. (2020). ECG-derived respiratory rate in atrial fibrillation. IEEE Trans. Biomed. Eng. 67, 905–914. 10.1109/TBME.2019.2923587 31226064

[B31] Lian J. Müssig D. Lang V. (2006). Computer modeling of ventricular rhythm during atrial fibrillation and ventricular pacing. IEEE Trans. Biomed. Eng. 53, 1512–1520. 10.1109/TBME.2006.876627 16916085

[B32] Linz D. Elliott A. D. Hohl M. Malik V. Schotten U. Dobrev D. (2019). Role of autonomic nervous system in atrial fibrillation. Int. J. Cardiol. 287, 181–188. 10.1016/j.ijcard.2018.11.091 30497894

[B33] Lister J. W. Stein E. Kosowsky B. D. Lau S. H. Damato A. N. (1965). Atrioventricular conduction in man: effect of rate, exercise, isoproterenol and atropine on the P-R interval. Am. J. Cardiol. 16, 516–523. 10.1016/0002-9149(65)90028-7 5834472

[B34] Loecher M. Perotti L. E. Ennis D. B. (2021). Using synthetic data generation to train a cardiac motion tag tracking neural network. Med. Image Anal. 74, 102223. 10.1016/j.media.2021.102223 34555661 PMC8560564

[B35] Malik V. Elliott A. D. Thomas G. Mishima R. S. Pitman B. Middeldorp M. E. (2022). Autonomic afferent dysregulation in atrial fibrillation. JACC Clin. Electrophysiol. 8, 152–164. 10.1016/j.jacep.2021.10.010 35210071

[B36] Mangin L. Vinet A. Pagé P. Glass L. (2005). Effects of antiarrhythmic drug therapy on atrioventricular nodal function during atrial fibrillation in humans. Europace 7, S71–S82. 10.1016/j.eupc.2005.03.016 16102505

[B37] Marmerstein J. T. McCallum G. A. Durand D. M. (2021). Direct measurement of vagal tone in rats does not show correlation to HRV. Sci. Rep. 11, 1210. 10.1038/s41598-020-79808-8 33441733 PMC7807082

[B38] Martin P. (1975). Dynamic vagal control of atrial-ventricular condition: theoretical and experimental studies. Ann. Biomed. Eng. 3, 275–295. 10.1007/BF02390973 1220583

[B39] Masè M. Marini M. Disertori M. Ravelli F. (2015). Dynamics of AV coupling during human atrial fibrillation: role of atrial rate. Am. J. Physiol. Heart Circ. Physiol. 309, H198–H205. 10.1152/ajpheart.00726.2014 25910809

[B40] Miyoshi M. Kondo H. Ishii Y. Shinohara T. Yonezu K. Harada T. (2020). Baroreflex sensitivity in patients with atrial fibrillation. J. Am. Hear. Assoc. 9, e018019. 10.1161/jaha.120.018019 PMC7955376 33263265

[B41] Nagayoshi H. Janota T. Hnatkova K. Camm A. J. Malik M. (1997). Autonomic modulation of ventricular rate in atrial fibrillation. Am. J. Physiol. Heart Circ. Physiol. 272, H1643–H1649. 10.1152/ajpheart.1997.272.4.H1643 9139946

[B42] Nayebpour M. Talajic M. Villemaire C. Nattel S. (1990). Vagal modulation of the rate-dependent properties of the atrioventricular node. Circ. Res. 67, 1152–1166. 10.1161/01.RES.67.5.1152 2171801

[B43] Ottaviani M. M. Wright L. Dawood T. Macefield V. G. (2020). *In vivo* recordings from the human vagus nerve using ultrasound-guided microneurography. J. Physiol. 598, 3569–3576. 10.1113/JP280077 32538473

[B44] Pacchia C. F. Kline G. P. Hamdan M. H. Clark K. G. Clark M. G. Smith M. L. (2011). Oscillatory vagal maneuvers produce ventricular entrainment in patients with atrial fibrillation. Clin. Auton. Res. 21, 325–332. 10.1007/s10286-011-0117-7 21553203

[B45] Patros M. Ottaviani M. M. Wright L. Dawood T. Macefield V. G. (2022). Quantification of cardiac and respiratory modulation of axonal activity in the human vagus nerve. J. Physiol. 600, 3113–3126. 10.1113/JP282994 35524982

[B46] Piepoli M. Sleight P. Leuzzi S. Valle F. Spadacini G. Passino C. (1997). Origin of respiratory sinus arrhythmia in conscious humans. An important role for arterial carotid baroreceptors. Circ 95, 1813–1821. 10.1161/01.CIR.95.7.1813 9107168

[B47] Pintea S. L. Lin Y. Dijkstra J. van Gemert J. C. (2023). A step towards understanding why classification helps regression. 2023 IEEE/CVF Int. Conf. Comput. Vis. (ICCV), 19915–19924. 10.1109/ICCV51070.2023.01828

[B48] Pirola F. T. Potter E. K. (1990). Vagal action on atrioventricular conduction and its inhibition by sympathetic stimulation and neuropeptide Y in anaesthetised dogs. J. Auton. Nerv. Syst. 31, 1–12. 10.1016/0165-1838(90)90166-g 2262662

[B49] Pitzalis M. V. Massari F. Forleo C. Fioretti A. Colombo R. Balducci C. (1999). Respiratory systolic pressure variability during atrial fibrillation and sinus rhythm. Hypertension 34, 1060–1065. 10.1161/01.HYP.34.5.1060 10567182

[B50] Plappert F. Wallman M. Abdollahpur M. Platonov P. G. Östenson S. Sandberg F. (2022). An atrioventricular node model incorporating autonomic tone. Front. Physiol. 13, 976468. 10.3389/fphys.2022.976468 36187793 PMC9520409

[B51] Priola D. V. Curtis M. B. Anagnostelis C. Martinez E. (1983). Altered nicotinic sensitivity of AV node in surgically denervated canine hearts. Am. J. Physiol. 245, 27–32. 10.1152/ajpheart.1983.245.1.H27 6869561

[B52] Rashidi A. Khodarahmi I. (2005). Nonlinear modeling of the atrioventricular node physiology in atrial fibrillation. J. Theor. Biol. 232, 545–549. 10.1016/j.jtbi.2004.08.033 15588634

[B53] Rawles J. M. Pai G. R. Reid S. R. (1989). Paradoxical effect of respiration on ventricular rate in atrial fibrillation. Clin. Sci. 76, 109–112. 10.1042/cs0760109 2920528

[B54] Russo M. A. Santarelli D. M. O’Rourke D. (2017). The physiological effects of slow breathing in the healthy human. Breathe (Sheff) 13, 298–309. 10.1183/20734735.009817 29209423 PMC5709795

[B55] Sameni R. Jutten C. Shamsollahi M. B. (2008). Multichannel electrocardiogram decomposition using periodic component analysis. IEEE Trans. Biomed. Eng. 55, 1935–1940. 10.1109/TBME.2008.919714 18632355

[B56] Sassi R. Cerutti S. Lombardi F. Malik M. Huikuri H. V. Peng C.-K. (2015). Advances in heart rate variability signal analysis: joint position statement by the e-cardiology ESC working group and the European heart rhythm association co-endorsed by the asia pacific heart rhythm society. Europace 17, 1341–1353. 10.1093/europace/euv015 26177817

[B57] Shaffer F. Ginsberg J. P. (2017). An overview of heart rate variability metrics and norms. Front. Public Health 5, 258. 10.3389/fpubh.2017.00258 29034226 PMC5624990

[B58] Shen M. J. Zipes D. P. (2014). Role of the autonomic nervous system in modulating cardiac arrhythmias. Circ. Res. 114, 1004–1021. 10.1161/CIRCRESAHA.113.302549 24625726

[B59] Smith L. N. (2017). Cyclical learning rates for training neural networks. 2017 IEEE Winter Conf. Appl. Comput. Vis. (WACV), 464–472. 10.1109/WACV.2017.58

[B60] Sološenko A. Paliakaitė B. Marozas V. Sörnmo L. (2022). Training convolutional neural networks on simulated photoplethysmography data: application to bradycardia and tachycardia detection. Front. Physiol. 13, 928098. 10.3389/fphys.2022.928098 35923223 PMC9339964

[B61] Spear J. F. Moore E. N. (1973). Influence of brief vagal and stellate nerve stimulation on pacemaker activity and conduction within the atrioventricular conduction system of the dog. Circ. Res. 32, 27–41. 10.1161/01.RES.32.1.27 4684126

[B62] Stridh M. Sörnmo L. (2001). Spatiotemporal QRST cancellation techniques for analysis of atrial fibrillation. Ieee. Trans. Biomed. Eng. 48, 105–111. 10.1109/10.900266 11235581

[B63] Świt P. Pollap A. Orzeł J. (2023). Spectroscopic determination of acetylcholine (ACh): a representative review. Top. Curr. Chem. 381, 16. 10.1007/s41061-023-00426-9 PMC10175388 37169979

[B64] Tan A. Y. Zhou S. Ogawa M. Song J. Chu M. Li H. (2008). Neural mechanisms of paroxysmal atrial fibrillation and paroxysmal atrial tachycardia in ambulatory canines. Circ 118, 916–925. 10.1161/CIRCULATIONAHA.108.776203 PMC2742977 18697820

[B65] Trayanova N. A. Popescu D. M. Shade J. K. (2021). Machine learning in arrhythmia and electrophysiology. Circ. Res. 128, 544–566. 10.1161/CIRCRESAHA.120.317872 33600229 PMC7899082

[B66] van den Berg M. P. Hassink R. J. Tuinenburg A. E. van Sonderen E. F. L. P. Lefrandt J. D. de Kam P. J. (2001). Quality of life in patients with paroxysmal atrial fibrillation and its predictors: importance of the autonomic nervous system. Eur. Hear. J. 22, 247–253. 10.1053/euhj.2001.2180 11161936

[B67] Varon C. Morales J. Lázaro J. Orini M. Deviaene M. Kontaxis S. (2020). A comparative study of ECG-derived respiration in ambulatory monitoring using the single-lead ECG. Sci. Rep. 10, 5704. 10.1038/s41598-020-62624-5 32235865 PMC7109157

[B68] Wallman M. Sandberg F. (2018). Characterisation of human AV-nodal properties using a network model. Med. Biol. Eng. Comput. 56, 247–259. 10.1007/s11517-017-1684-0 28702812

[B69] Wang D. Veltmann C. Bauersachs J. Duncker D. (2023). Antiarrhythmic effects of baroreceptor activation therapy in chronic heart failure: a case report. Eur. Hear. J. - Case Rep. 7, ytad520. 10.1093/ehjcr/ytad520 PMC10633707 37954563

[B70] Wasmund S. L. Li J.-M. Page R. L. Joglar J. A. Kowal R. C. Smith M. L. (2003). Effect of atrial fibrillation and an irregular ventricular response on sympathetic nerve activity in human subjects. Circ 107, 2011–2015. 10.1161/01.cir.0000064900.76674.cc 12681998

[B71] Weine J. van Gorkum R. J. Stoeck C. T. Vishnevskiy V. Kozerke S. (2022). Synthetically trained convolutional neural networks for improved tensor estimation from free-breathing cardiac DTI. Comput. Med. Imaging Graph. 99, 102075. 10.1016/j.compmedimag.2022.102075 35636378

[B72] Yu Y. Wei C. Liu L. Lian A. L. Qu X. F. Yu G. (2014). Atrial fibrillation increases sympathetic and parasympathetic neurons in the intrinsic cardiac nervous system. Pacing Clin. Electrophysiol. 37, 1462–1469. 10.1111/pace.12450 25053212

