# Supplementary material for: ECG-based estimation of respiration-induced autonomic modulation of AV nodal conduction during atrial fibrillation
Source: Front Physiol. 2024 May 8;15:1281343. doi: 10.3389/fphys.2024.1281343 (PMC11110927; doi:10.3389/fphys.2024.1281343)
Supplement: Supplementary file 1 [file DataSheet1.pdf]

Supplementary Material

1 ESTIMATION OF RESPIRATORY MODULATION

1.1 Training and estimation of respiratory modulation using a support vector machine model

A support vector machine (SVM) regression model is used here to estimate the peak-to-peak amplitude of respiration-induced autonomic modulation a_{resp}. The SVM $\mathcal{M}_{RR,Resp,AFR}$ was trained using a training dataset $\boldsymbol{\mathcal{X}}^{Sim,Train}$ with the format $\boldsymbol{\mathcal{X}} = [\mathcal{X}_{RR}^{Sim}; \mathcal{X}_{Resp}^{Sim}; \mathcal{X}_{AFR}^{Sim}]$ containing 100 000 parameter sets, as described in Sec. 2.3.2. The SVM was trained with the MATLAB function fitrsvm (MATLAB 2023a) with a Gaussian kernel and normalized predictor data with zero mean and unit variance. The performance of $\mathcal{M}_{RR,Resp,AFR}$ on simulated data was assessed using the testing dataset $\boldsymbol{\mathcal{X}}^{Sim,Test}$ containing 2 million parameter sets, as described in Sec. 2.3.2 of the main manuscript. The performance on $\boldsymbol{\mathcal{X}}^{Sim,Test}$ was assessed using the RMSE, Pearson correlation, and coefficient of determination R^2 between the true a_{resp} and estimated $\hat{a}_{resp}$.

1.2 Accuracy of support vector machine

The distribution of estimated $\hat{a}_{resp}$ over true a_{resp} for $\mathcal{M}_{RR,Resp,AFR}$ is shown in Fig. S1. Also displayed in Fig. S1 for comparison based on the same data are the corresponding distributions for estimation using a

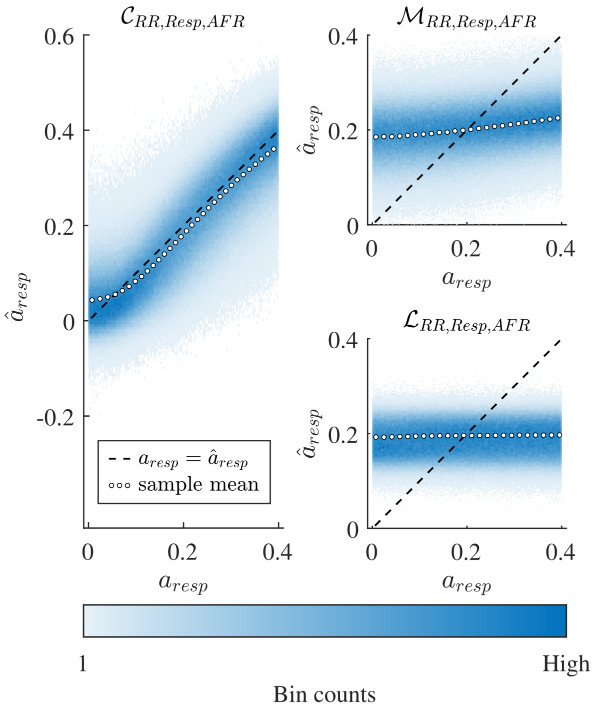

Figure S1. Binned scatter plot of estimated $\hat{a}_{resp}$ versus true a_{resp} for the CNN $\mathcal{C}_{RR,Resp,AFR}$, support vector machine $\mathcal{M}_{RR,Resp,AFR}$ and linear regression $\mathcal{L}_{RR,Resp,AFR}$, where all three were based on the same input data $\boldsymbol{\mathcal{X}} = [\mathcal{X}_{RR}^{Sim}; \mathcal{X}_{Resp}^{Sim}; \mathcal{X}_{AFR}^{Sim}]$. The black dotted line shows where $\hat{a}_{resp}$ is equal to a_{resp}. The white dotted line shows the sample mean of the $\hat{a}_{resp}$ estimation.

Table S1. RMSE, Pearson sample correlation and R^2 of $\mathcal{C}_{RR,Resp,AFR}$, support vector machine $\mathcal{M}_{RR,Resp,AFR}$ and linear regression $\mathcal{L}_{RR,Resp,AFR}$ using 1-minute segments.

	RMSE	Pearson correlation r	R^2
$\mathcal{C}_{RR,Resp,AFR}$	0.066	0.855	0.674
$\mathcal{M}_{RR,Resp,AFR}$	0.113	0.254	0.036
$\mathcal{L}_{RR,Resp,AFR}$	0.119	0.037	-0.068

1-dimensional convolutional neural network $\mathcal{C}_{RR,Resp,AFR}$ and linear regression $\mathcal{L}_{RR,Resp,AFR}$, described in Sec. 2.4.1 and 2.4.2 of the main manuscript. The RMSE, Pearson sample correlation and R^2 are listed for $\mathcal{C}_{RR,Resp,AFR}$, $\mathcal{M}_{RR,Resp,AFR}$, and $\mathcal{L}_{RR,Resp,AFR}$ in Table S1. The $\mathcal{L}_{RR,Resp,AFR}$ was unable to estimate a_{resp} (Pearson sample correlation $r = 0.037$); the $\mathcal{M}_{RR,Resp,AFR}$ was able to do some adaptation to the data ($r = 0.254$); however, both $\mathcal{L}_{RR,Resp,AFR}$ and $\mathcal{M}_{RR,Resp,AFR}$ performed clearly worse in estimating a_{resp} than the investigated CNN $\mathcal{C}_{RR,Resp,AFR}$ ($r = 0.855$).
